# High quality de novo genome assembly of the non-conventional yeast *Kazachstania bulderi* describes a potential low pH production host for biorefineries

**DOI:** 10.1038/s42003-023-05285-0

**Published:** 2023-09-07

**Authors:** Laura N. Balarezo-Cisneros, Soukaina Timouma, Alistair Hanak, Andrew Currin, Fernando Valle, Daniela Delneri

**Affiliations:** 1 Manchester Institute of Biotechnology, University of Manchester, Manchester, UK; 2 BP Biosciences Center, San Diego, CA USA

**Keywords:** Industrial microbiology, Genome

## Abstract

*Kazachstania bulderi* is a non-conventional yeast species able to grow efficiently on glucose and δ-gluconolactone at low pH. These unique traits make *K. bulderi* an ideal candidate for use in sustainable biotechnology processes including low pH fermentations and the production of green chemicals including organic acids. To accelerate strain development with this species, detailed information of its genetics is needed. Here, by employing long read sequencing we report a high-quality phased genome assembly for three strains of *K. bulderi* species, including the type strain. The sequences were assembled into 12 chromosomes with a total length of 14 Mb, and the genome was fully annotated at structural and functional levels, including allelic and structural variants, ribosomal array and mating type locus. This high-quality reference genome provides a resource to advance our fundamental knowledge of biotechnologically relevant non-conventional yeasts and to support the development of genetic tools for manipulating such strains towards their use as production hosts in biotechnological processes.

## Abstract (web summary)

A comprehensive genome assembly from three strains of the yeast, *Kazachstania bulderi*, serves as a resource to explore the biotechnological potential of this yeast, particularly as a low pH production host for biorefineries.

## Introduction

Biorefineries have been proposed as a solution to replace oil-derived products with more sustainable biotechnologies, based on the use of renewable resources in the commercial production of chemicals and other products. These types of processes are often hampered by the high costs associated with the recovery and purification of the products, which in many cases, can be as high as 50–80% of the total production cost^1–3^. This is particularly evident for the production of organic acids by fermentation. Because most production microorganisms used in the industry need a neutral pH for optimal performance during the fermentation process, as the organic acids are synthesized and accumulated in the culture media, pH decreases. The organic acid, then, has to be neutralized by the addition of a base, leading to the formation of the organic acid salt^4,5^. Subsequent applications of organic acids normally use the acid form, and the neutralizing cation has to be removed, disposed or recycled^6^. There is, therefore, a need to develop new production hosts that have an optimum performance at low pH. Production of organic acids at low pH, would decrease or eliminate the formation of the organic salts, and improve product recovery and overall process economics. Three strains of *Kazachstania bulderi*, formerly *Saccharomyces bulderi*, isolated from maize silage^7^, have been reported to rapidly and efficiently, ferment glucose and δ-gluconolactone to ethanol and carbon dioxide at pH between 2.5 and 5.0^8^. Furthermore, *K. bulderi* has also been isolated from French, Belgian, Spanish, and Turkish sourdoughs^9–14^. These characteristics make *K. bulderi* a very attractive host for low-pH organic acid production. However, the development of a non-conventional yeast as a potential production host for commercial processes requires a deep understanding of its genomic sequence and organization. Additionally, accurate gene annotations and the development of bespoke genetic tools are needed to modify the strain and create the desired phenotypes.

*Kazachstania*, and their close neighbors, *Naumovozyma* and *Saccharomyces* genera, arose after the whole-genome duplication event within *Saccharomycetaceae*^15^. The *Kazachstania* genus encompasses a large and diverse group of ascomycetous budding yeasts. To date, it is composed of over 40 species isolated from wild, domesticated and clinical environments^7,16^.

Currently, high quality genome assemblies at chromosome level for *Kazachstania* strains are still limited. Presently, only four assembled and annotated genomes have been published, for *K. africana*^17^, *K. naganishii*^17^, *K. saulgeensis*^18^, and *K. barnettii*^16^. Highly fragmented assemblies have been drafted for seven species, including *K. humilis*^19^*, K. servazzii*^20,21^, *K. telluris*^22^, *K. slooffiae*^23^*, K. bovina*^24^, *K. exigua*, and *K. unispora*^23^. A fragmented genome for *K. bulderi* CBS 8638 has been deposited in NCBI (PRJEB44438). Other *Kazachstania* species have a draft assembly with very few, or no annotations^25,26^.

Here, we assessed the ability of these strains to tolerate organic acids to contextualize their biotechnological potential. Next, using a combination of next generation sequencing, de novo assembly tools and gene annotation algorithms, we construct high-quality reference genomes, including ribosomal repeats, the mating type locus, and mitochondria DNA for the three reported *K. bulderi* strains CBS 8638 (type strain), CBS 8639 and NRRL Y-27205. We carried out comparative genomic analysis of the strains, including allelic and structural variation, phylogenetic analysis, synteny, and identification of species-specific and genus-specific genes. The availability of this genome will facilitate gene manipulation tools for *K. bulderi* and its development as a production host for sustainable green products. This will also provide a platform for future population genomic, or ‘omics studies for other *Kazachstania species*.

## Results and discussion

### Phenotypic characteristics of *K. bulderi* strains at low pH, organic acids, and antimicrobial drugs

Since *K. bulderi* was isolated, only two studies have been conducted to investigate its physiological characteristics (7, 8). This species was shown to be able to grow efficiently at low pHs ranging from 5.0 to 2.5 (8). As a primary step in conducting this comprehensive genome study, we evaluated the biotechnological potential of these strains as low pH hosts, by assessing their pH range of growth, their tolerance to organic acids of industrial interest, and their resistance to antimicrobial drugs, important markers for future genetic manipulations.

We showed that the three *K. bulderi* strains CBS 8638, CBS 8639, and NRRL Y-27205 exhibited high growth rate, at pH as low as 2.1, with CBS 8638, showing the strongest growth (Supplementary Fig. 1 and Supplementary Tables 1, 2).

The *K. bulderi* strains also grew in the presence of different concentrations of lactic acid (Supplementary Fig. 2a–f) and in 25 mM formic acid (Supplementary Fig. 2g). In the presence of 85 g/L lactic acid, CBS 8638 was the only strain that consistently maintained optimal growth, while *S. cerevisiae* BY4743 did not grow (Supplementary Fig. 2e, Supplementary Table 2). In the presence of 1 g/L acrylic acid, none of the strains grew, highlighting the high toxicity of this organic acid even at low concentrations (Supplementary Fig. 2h). All growth parameters and biomass yield for the strains are reported in Supplementary Tables 1 and 2, respectively. These results highlight the ability of CBS 8638 and CBS 8639 strains to maintain growth under challenging environmental conditions, which could have important implications for their potential use in various industrial applications.

We evaluated the resistance of *K. bulderi* strains to common antimicrobial drugs used in the laboratory as selection markers to inform the development of genetic tools and to guide practical genetic manipulation methodologies for this species.

We observed that CBS 8638 and CBS 8639 were resistant to high concentrations of hygromycin B and phleomycin, when compared to three *S. cerevisiae* strains, namely the lab strain BY4741, the commercial strain NCYC 505, and the natural strain 96.2 (Supplementary Fig. 3). Among the *K. bulderi* strains, NRRL Y-27205 showed the least resistance to these drugs. Such results suggested a reduced cellular uptake of cationic drugs in this species, and it is therefore important to increase the common working concentrations of hygromycin B and phleomycin in marker selection experiments. Nourseothricin was the most effective drug at growth inhibition for all *K. bulderi* strains, followed by G418 (Supplementary Fig. 3), and was the most suitable candidate as a marker for genetic engineering in this species.

### Genome sequencing and de novo assembly of the three *K. bulderi* strains

**Fig. 1:**
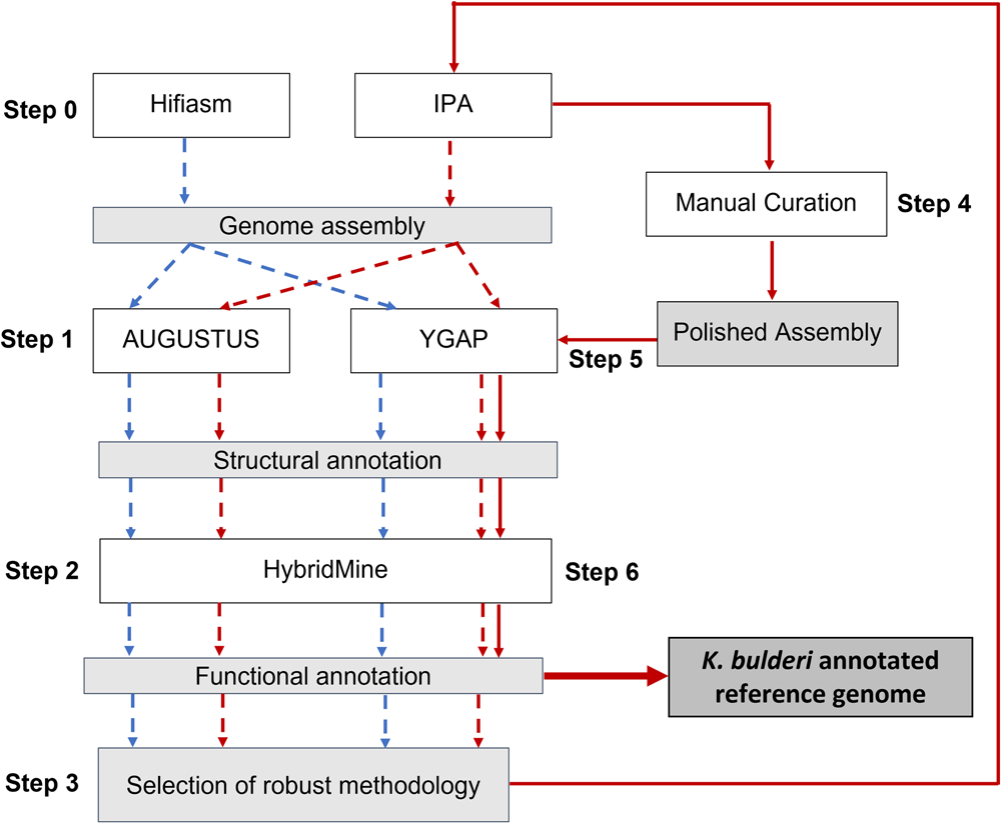
Workflow for generating high-quality genome assembly of *K. bulderi* strains. Preliminary genome assembly (dashed arrows), including assemblers (IPA and HIFIASM), structural annotation algorithms (YGAP and AUGUSTUS), and the functional annotation tool (HybridMine), was performed to select the most robust genome assembly methodology. The final pipeline (continuous arrows) includes the IPA assembly, the manual curation, and the structural (YGAP) and functional annotation (HybridMine) to generate the final *K. bulderi* genome reference. Methodologies are depicted by white rectangular boxes and outputs by gray rectangular boxes.

Genome sequencing of *K. bulderi* CBS 8638, CBS 8639, and NRRL- Y27205 strains was performed using HiFi read data derived from single-molecule real-time (SMRT) technology from Pacific Biosciences (PacBio), the de novo phased assembly and annotation strategy is summarized in Fig. 1.

We obtained ~131,888, 125,890, and 159,349 reads for CBS 8638, CBS 8639 and NRRL Y-27205 respectively. To ensure a high-quality genome assembly we tested two different phased assembly algorithms, the Improved Phased Assembler (IPA; the official PacBio software for HiFi genome assembly) and HIFIASM (a fast haplotype-resolved de novo assembler for PacBio HiFi reads) on *K. bulderi* CBS 8638, and CBS 8639 strains. The IPA assembler consistently gave a better assembly with a number of contigs closer to the number of chromosomes found in other *Kazachstania* species^16–18^, while HIFIASM assembler generated a much higher number of contigs for CBS 8638 and CBS 8639 (Supplementary Table 3).

IPA generated primary de novo assemblies of 14, 17, and 15 contigs for CBS 8638, CBS 8639 and NRRL-Y27205 respectively, totaling ca. 14 Mb in length (Supplementary Table 3). It also generated alternative haplotig assemblies of 85, 108 and 172 contigs for CBS 8638, CBS 8639 and NRRL Y-27205, respectively (Supplementary Table 3). These alternative haplotigs represent regions of heterozygosity, which allow separation of haplotypes for *K. bulderi* diploid strains, and spanned a total of 13 Mb, 14 Mb, and 16 Mb (100% was separated into haplotypes). The total coverage was 59X for CBS 8638, 63Xfor CBS 8639, and 83.9X for NRRL Y-27205 (Supplementary Table 4). Overall, the alternative and primary assemblies share more than 92% of structural elements and show collinearity (Supplementary Table 5; Supplementary Fig. 4).

A high-quality assembly is determined when it respects three crucial attributes, referred as the three “C”s^27^: Continuous (size of contigs), Correct (how well the contigs actually represent the genome sequence) and Complete (ability to complete the whole structure of the genome). The assemblies obtained using the IPA assembler were selected because they best fulfill the two criteria of continuity and completeness.

To select for the assembly methodology which give the best mapping of genetic elements, we then carried out a preliminary annotation using both AUGUSTUS^28^ and YGAP^29^, and the predicted proteins were functionally annotated using HybridMine (Fig. 1). AUGUSTUS uses a hidden Markov model (probabilistic approach) to predict structural elements, whereas YGAP uses synteny ancestry (homology approach). HybridMine predicts one-to-one orthologs^30^ to infer function, and predicts groups of homologs, including paralogs, using a well annotated reference species, such as *S. cerevisiae*. We observed that the structural annotation method that consistently produced higher number of functionally annotated genes was YGAP. AUGUSTUS predicted a higher number of genes and proteins compared to YGAP (Supplementary Fig. 5a), however, when analyzed with HybridMine, it also had a higher number non functionally annotated proteins, suggesting that they are either not real genes, or the protein sequence was not well predicted, or not translated. As expected, YGAP performed better here given that it is optimized towards yeast genome structural annotation. YGAP is capable of efficiently inferring introns/exons, therefore, protein sequences can be more accurately predicted^29^.

HybridMine was able to functionally annotate more than 90% of protein-coding genes regardless of the method used (Supplementary Fig. 5b).

The assembly generated using the IPA assembler (Fig. 1) was used as starting point for manual curation.

When comparing assemblies across individual *K. bulderi* strains, it was clear that: *i*. in one *K. bulderi* strain, certain primary contigs were fragmented into either two or three separate contigs (Supplementary Table 6); *ii*. across the strains, there were regions with non-uniform distribution in the mapping of long reads versus the assemblies; *iii*. translocated and inverted sequences were identified within the middle of specific contigs.

To address these complexities, manual curation was conducted. This involved analyzing the alignment of the three *K. bulderi* assemblies and the mapping of HiFi reads against each assembly. Contigs that had initially been divided in different strains were meticulously validated and consolidated through PCR (Supplementary Note 1, Supplementary Fig. 6), resulting in a total of 12 contigs across all strains.

Regions lacking read coverage were addressed using the alternative contigs of each strain individually. This approach enabled the recovery of a fragment containing 56 genes on chromosome V of the CBS 8638 genome, a segment that had been lost during the initial genome assembly (Supplementary Note 2, Supplementary Fig. 7). Additionally, this detailed curation unveiled the translocation as an artifact arising from a misassembly (Supplementary Note 3, Supplementary Fig. 8). The inversion between CBS 8639 and NRRL Y-27205 was further confirmed through experimental validation using PCR.

Following manual curation, reads were re-mapped to their respective assemblies resulting in a regular and uniform reads coverage. The primary assemblies now consisted of 12 contigs, totaling 14 Mb, with contig N50 of 1.2 Mb (Table 1). The coverage of the manually curated assemblies increased to 62X, 65X, and 84X for CBS 8638, CBS 8639, and NRRL Y-27205, respectively (Table 1) and a chromosome- level assembly was achieved (Table 2). *K. bulderi* CBS 8638, CBS 8639, and NRRL Y-27205 chromosome number and size was also confirmed via pulse field gel electrophoresis (Supplementary Fig. 9).

**Table 1:** Metrics and summary statistics for the curate *K. bulderi* de novo PacBio assemblies.

Metric	De novo assemblies		
	CBS 8638	CBS 8639	NRRL-Y27205
Size (Mb)	14.47	14.25	14.44
N° Contigs	12	12	12
Largest contig (bp)	2767906	2786749	2758527
N50 (Mb)	1.25	1.24	1.23
N75 (Mb)	0.84	0.86	0.90
L50	4	4	4
L75	7	7	7
GC (%)	33.12	33.11	33.16
Average identity (%)	97.5	97.3	97.3
Mean read length	6042.8	6607.6	7153.3
Median identity (%)	99.4	99.3	99.3
Median read length	5871	6744	7308
Number of reads	149370	136974	167910
Read length N50	7933	8148	8920
Total bases	902618975	905075306	1201106262
Total bases aligned	843227356	879022084	1162715498
Coverage (X)	62.4	64.5	83.9

**Table 2:** Total length of chromosome-level genome assembly for three *K. bulderi* strains after curation.

Chromosome	CBS 8638	CBS 8639	NRRL Y-27205
	Size (Mb)		
Chr I	2.79	2.77	2.76
Chr II	2.72	2.73	2.72
Chr III	1.42	1.37	1.43
Chr IV	1.24	1.25	1.22
Chr V	1.12	1.18	1.23
Chr VI	0.99	0.94	1.01
Chr VII	0.86	0.78	0.90
Chr VIII	0.80	0.80	0.84
Chr IX	0.75	0.73	0.73
Chr X	0.72	0.71	0.65
Chr XI	0.43	0.43	0.55
Chr XII	0.40	0.40	0.40

The completeness of the assemblies was also evaluated using the BUSCO software using the “-*Saccharomycetes*” data set of BUSCO gene collection^31^. Results indicated that 99% of the CBS 8638 and CBS 8639 assemblies and 98% of NRRL-Y27205 are complete (Supplementary Table 7). By comparison, the previous CBS 8639 and NRRL Y-27205 assembly had 97.6% and 97.3% complete BUSCO alignments respectively. The Missing BUSCO scores were 2.1% and 2.4% for both assemblies respectively, indicating a higher fraction of the genome was missing in both initial assemblies before curation (Supplementary Table 7).

### *K. bulderi* functional annotation

**Fig. 2:**
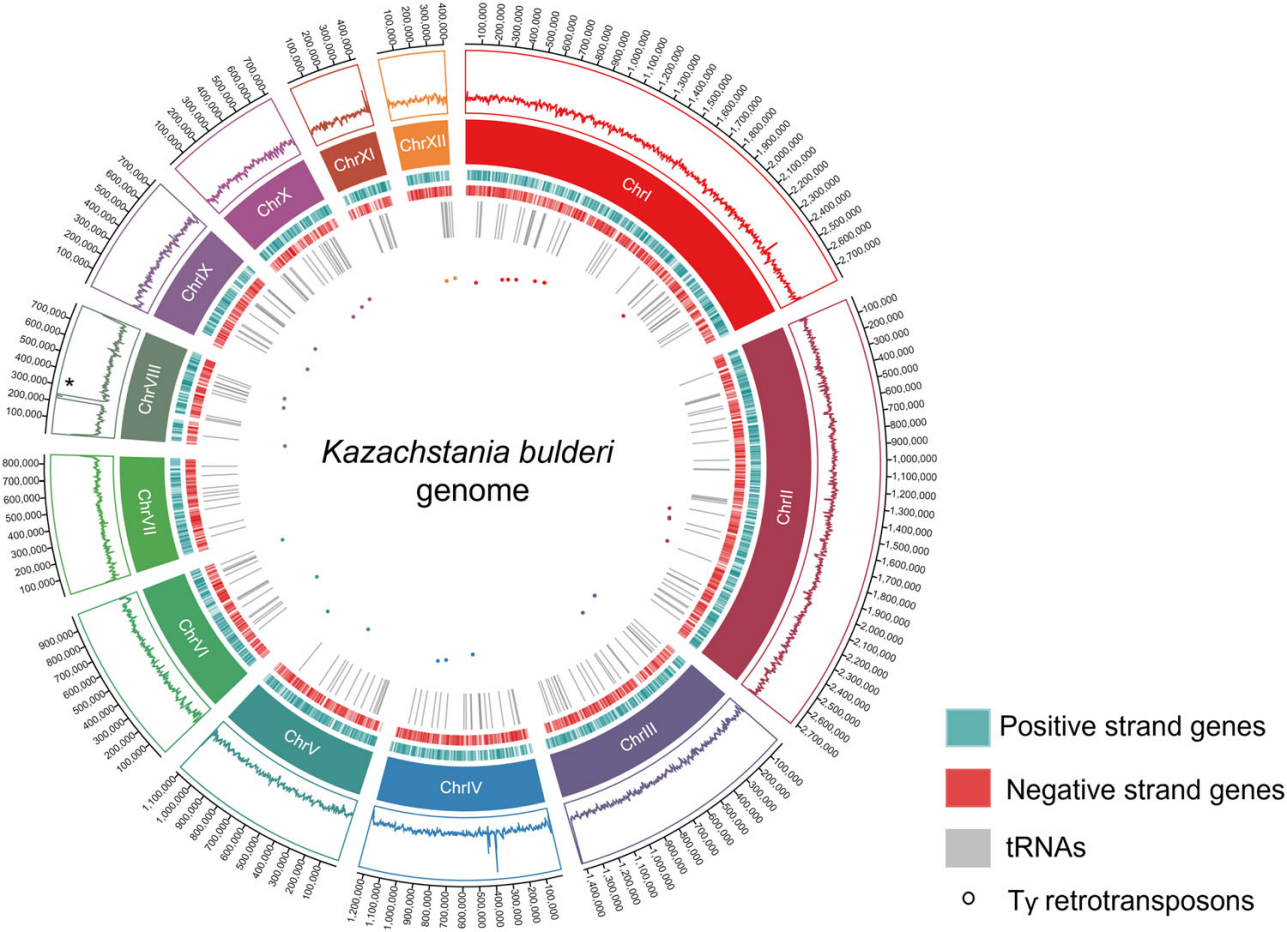
Graphical representation of chromosome-level scaffolds including annotated genomic elements for *K. bulderi*. The Circos Plot illustrates the annotation of genes, tRNAs, and transposable elements across 12 chromosome-level scaffolds. The inner-to-outer tracks feature Ty retrotransposons (circular dots), tRNAs (gray), genes on the positive strand (green), genes on the negative strand (red), distinct chromosome segments (color-coded), average read coverage (read depth track), and chromosome lengths. The asterisk on chromosome VIII highlights the peak of reads corresponding to rRNA repetitions.

YGAP identified a total of 5877, 5759, and 5769 structural elements for CBS 8638, CBS 8639, and NRRL Y-27205 assemblies after manual curation, respectively, including protein coding genes and tRNAs. Furthermore, Ty retrotransposons and rRNAs were also annotated (Fig. 2). The genomic features annotated per chromosome, in each strain are listed in Supplementary Table 8. The output of YGAP for the *K. bulderi* predicted genes used the following nomenclature: KB for *Kazachstania bulderi*; 38, 39, and Y27 for the strains CBS 8638, CBS 8639, and NRRL Y-27205, respectively. Consecutive alphabet letters were used for the chromosome number.

Functional annotation was inferred using the one-to-one orthologs found in the *S. cerevisiae* model yeast. As result, 4541, 4543, and 4523 proteins had a function inferred in *K. bulderi* CBS 8638, CBS 8639, and NRRL Y-27205, respectively. Additionally, groups of homolog proteins were also identified in each strain (Supplementary Data 1).

We searched for genes specific to *K. bulderi*, which could not be detected by using *S. cerevisiae* as a model organism. As *K. bulderi* was isolated from low pH environments, it is possible that these strains acquired or evolved several genes as an adaptation response. To identify any such genes, we first searched the one-to-one orthologs between *K. bulderi* and other model yeast species such as *Schizosaccharomyces pombe, Candida albicans, Candida glabrata* and *Yarrowia lipolytica* which are well functionally annotated. Secondly, we searched one-to-one orthologs in other species that grow well at low pH, such as *Kluyveromyces marxianus, Kluyveromyces lactis, Kazachstania exigua,* and *Kazachstania barnettii*, for which only structural annotations are available. In this way, we could expand the pool of annotated proteins, potentially including pH related genes, and detected genus-specific ones.

**Fig. 3:**
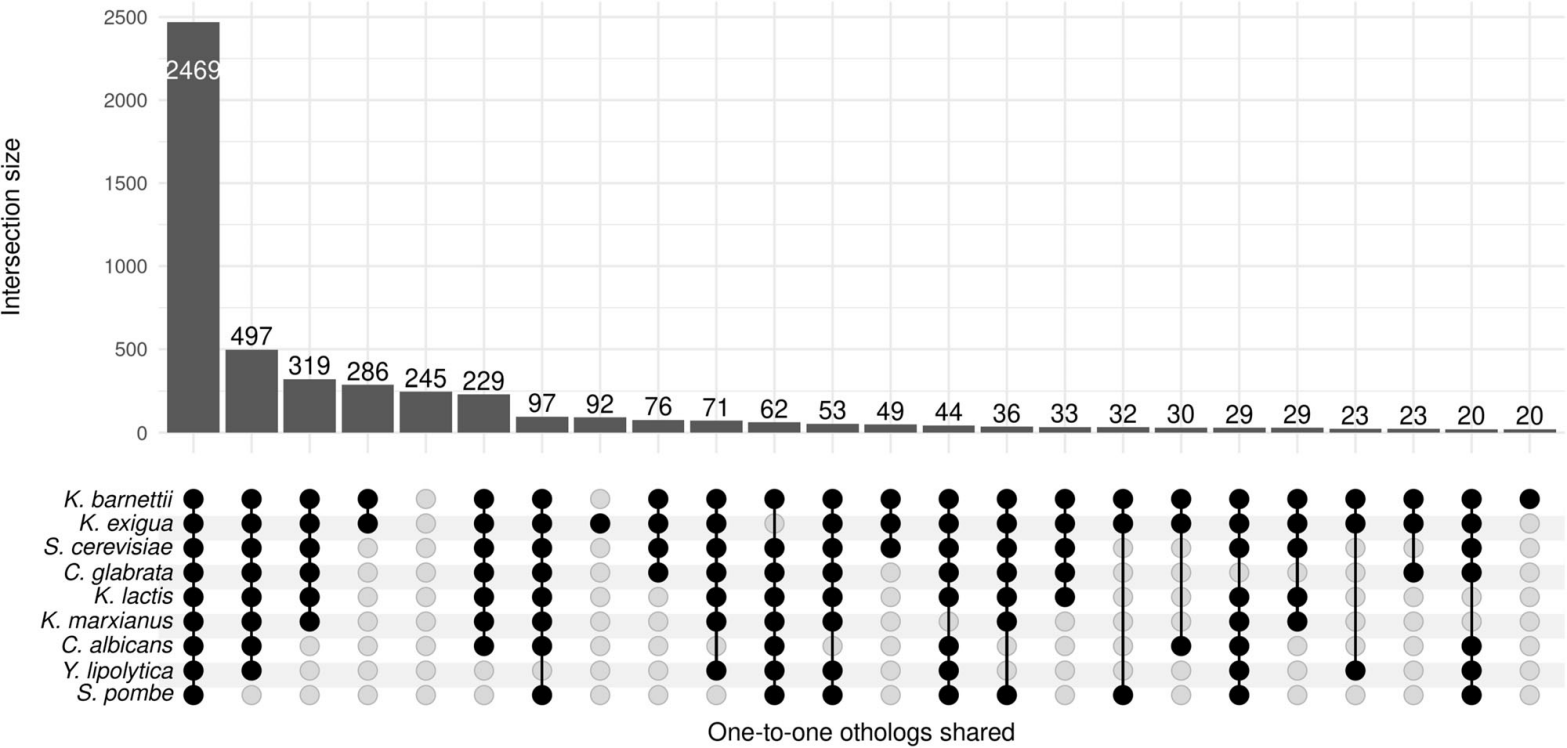
Comparative protein functional annotation in *K. bulderi* CBS 8639 using diverse reference genomes. Upset plot showing the number of proteins functionally annotated across *K. bulderi* CBS 8639 strain in common when using *Saccharomyces cerevisiae*, *Schizosaccharomyces pombe, Candida albicans, Candida glabrata, Yarrowia lipolytica*, *Kluyveromyces marxianus, Kluyveromyces lactis, Kazachstania exigua,* and *Kazachstania barnettii*, as references genomes. Vertical lines and dots across the species represent the proteins in common between the reference species and *K. Bulderi*.

As result, a total of 5385, 5306, and 5281 proteins for CBS 8638 (Supplementary Fig 10a, Supplementary Data 2), CBS 8639 (Fig. 3, Supplementary Data 2) and NRRL Y-27205 (Supplementary Fig 10b, Supplementary Data 2), respectively, were identified. These accounted for ~95% of all predicted proteins and had a one-to-one ortholog found in at least one of the model species used, thus, they are not *K. bulderi* specific (Fig. 3, Supplementary Table 9). As expected, the highest number of one-to-one orthologs were detected when comparing the predicted proteins with species from the *Kazachstania genus* (Supplementary Table 9, Supplementary Data 2). The 286 one-to-one orthologs between *K. bulderi* CBS 8639, *K. exigua,* and *K. barnettii* that are not shared with the other yeast species may point to genus specific elements (Supplementary Data 2).

Distinctly, 281, 245, and 281 (~5%) proteins of *K. bulderi* CBS 8638, CBS 8639, and NRRL Y-27205 do not have any one-to-one orthologs in any of the yeast species used. 126 genes are shared among the three strains (Supplementary Data 2) and therefore may be *K. bulderi* specific. Additionally, only, 193, 160, and 159 of these potential *K. bulderi* specific proteins, belong to a group of homologs predicted as younger paralogs by HybridMine (Supplementary Data 1).

Algorithms which use the primary sequence homology to assign functional annotation such as HybridMine do not work well on novel sequences in comparison to predictive models that use deep learning. Given these potential paralogs may have neo-functionalised or sub-functionalised we employed structure-based function prediction using deep learning, rather than homology-based method using a non-redundant protein database to predict function for the remaining 5% of genes.

Structure-based protein function prediction using graph convolutional networks has been shown to be very accurate in predicting the function of novel proteins^32^. We used the AlphaFold algorithm to predict the 3D structure of the potential *K. bulderi* specific proteins, and a graph convolutional network model, DeepFRI^33^, trained on protein structures and their associated GO terms, to predict Molecular Function (MF), Biological Process (BP) and Cellular Components (CC), GO terms and EC numbers from the protein structures. Of the potential *K. bulderi* specific proteins, 42, 40, and 3 had a protein structure predicted by Alphafold in CBS 8638, CBS 8639 and NRRL Y-27205, respectively. There were two predominant biological processes enriched for these proteins (Supplementary Data 3): RNA related processes and membrane transporters, suggesting these are rapidly diverging classes of proteins. This is in accordance with findings that across the whole tree of life, cytosolic proteins are under tight selection (i.e. they are needed for maintaining internal homeostasis), while membrane proteins are under strong adaptive selection. In fact, there are consistently fewer detectable orthologs for membrane proteins than for water-soluble ones^34^. Moreover, ribosomal proteins can present segments of high structural variations which are thought to help adaption to specific environments^35^.

To gain a better physiological insight on these species, we have annotated the glycolysis pathway including the main pyruvate pathways (Supplementary Fig. 11a) and the pentose phosphate pathway (PPE; Supplementary Fig. 12a).

The metabolic engineering strategies for enhancing for lactic acid production involve the deletion of pyruvate decarboxylase (*PDC*) or alcohol dehydrogenase (*ADH*) gene families to reduce ethanol accumulation and redirect the carbon flux from pyruvate to lactic acid^36^. We employed functional annotation to identify the enzymes and their homologs involved in the glycolysis, including the *PDC* and *ADH* gene groups, in all three *K. bulderi* strains (Supplementary Fig. 11). In contrast to *Saccharomyces cerevisiae*, which has three pyruvate decarboxylases (*PDC1, PDC5,* and *PDC6*), and five alcohol dehydrogenases (*ADH1-5*), *K. bulderi* strains have only two *ADH* genes, annotated as *ADH1*, and *ADH3*, and only one pyruvate decarboxylase gene, annotated as *PDC1*. However, NRRL Y-27205 strain contained two paralogs, annotated as *PDC1.1* and *PDC1.2*, while CBS 8638 and CBS 8639 have only one young paralog of *PDC1*, annotated as *PDC1.2*. These paralogs are not co-located or located next to *PDC1*, and are all sub-telomeric (i.e. within 30 kb from chromosome end) with the exception of *PDC1.2* in CBS8638 (Supplementary Table 10). These strains have evolved independently, and hence different patterns of gene loss and gene duplication of young paralogs could have occurred (Supplementary Fig 11b). Overall, there are a lower number of pyruvate decarboxylase and alcohol dehydrogenase homologs in *K. bulderi*, compared to *S. cerevisiae* (Supplementary Data 1), hence, a lower number of gene deletions would be needed in *K. bulderi* to remove these functions.

Our genomic data also shows that the glucose-6-phosphate dehydrogenase (*ZWF1*), a key enzyme of the PPE pathway, is expanded in *K. bulderi* CBS 8639 and NRRL Y-27205. Beside *ZWF1*, to two further homologs *ZWF1.1* and *ZWF1.2*, both located in sub-telomeric regions, were identified (Supplementary Fig 12b, Supplementary Data 1, Supplementary Table 10), hence, providing a rationale for the proficiency of *K. bulderi* to metabolize gluconolactone via selection on copy number (8). This genomic annotation provides valuable insights for future genetic engineering efforts in optimizing *K. bulderi* for desired biotechnological traits such as lactic acid production or bioethanol production.

### Structural identification between strains and experimental validation of detected rearrangements

**Fig. 4:**
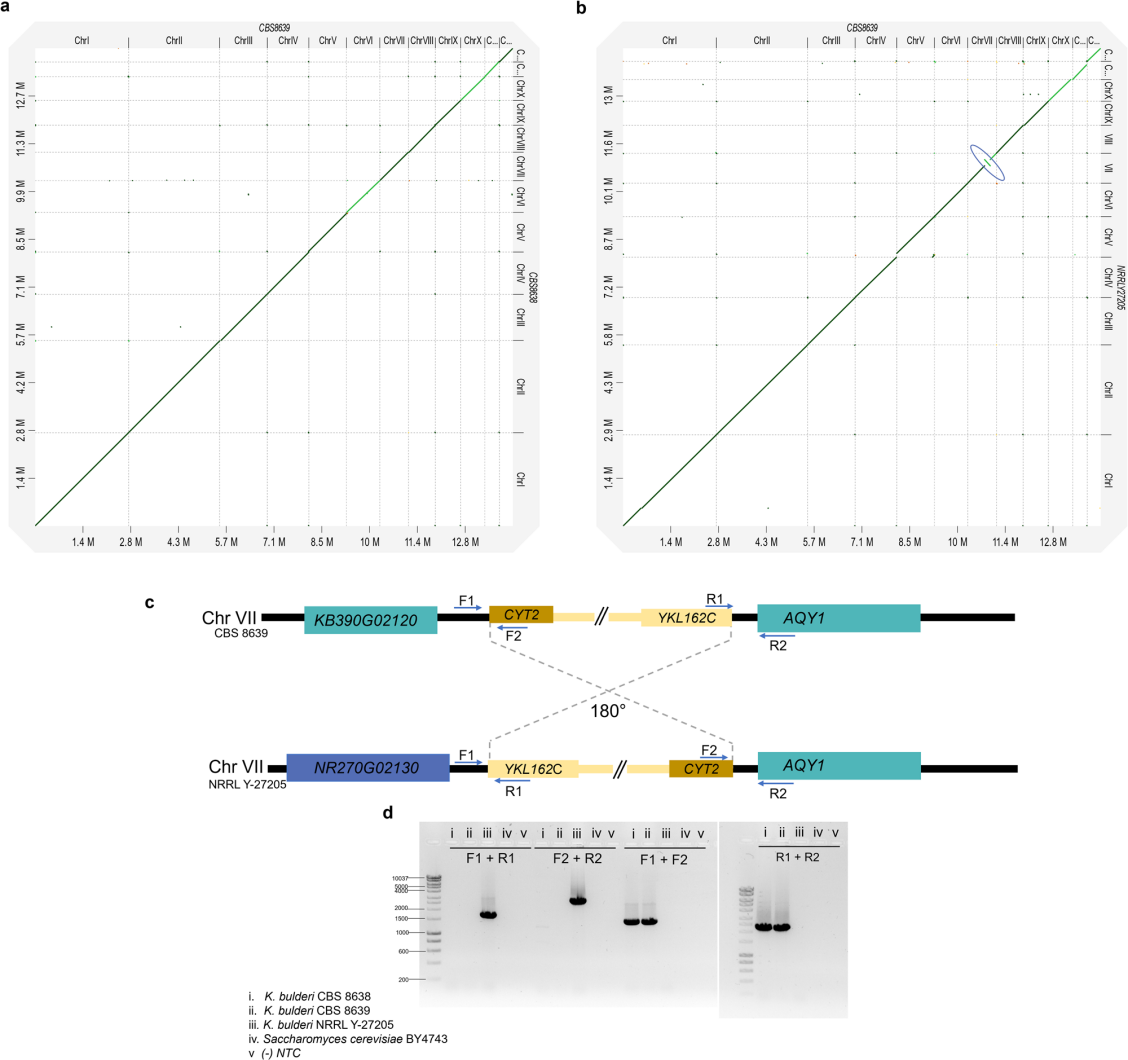
Identification of chromosomal rearrangements between *K. bulderi* strains. **a** Dot plot representing the alignment between *K. bulderi* CBS 8639 and CBS 8638 genomes. **b** Dot plot for the alignment between CBS 8639 and NRRL Y-27205 genomes. The inversion in chromosome VII for NRRL Y-27205 is highlighted in the blue circle. **c** Cartoon representation of the inversion including the flanking genes (not to scale) and the location of the primers used to confirm the inversion. **d** Gel electrophoresis of PCR products amplified to confirm the inversion. Different combinations of primers (F1-R1, F2-R2, F1-F2, and R1-R2) were used to amplify products in *K. bulderi* CBS 8638 (i), CBS 8639(ii) or NRRL Y-27205 (iii) and *S. cerevisiae* (iv) strains. The negative control has been carried out without DNA as template (v). In each case the resulting PCR products support the inversion identified by the genome assembly.

We compared our polished *K. bulderi* genome assemblies by considering the CBS 8639 strain as a reference outgroup. We found that all the 12 chromosomes of CBS 8638 are collinear with CBS 8639 (Fig. 4a), while NRRL Y-27205 presented a genomic rearrangement in chromosome VII. This region of about 167 kb, encompassing 71 genes, was inverted in NRRL Y-27205 genome compared to CBS 8639 (Fig. 4b).

We used diagnostic PCR to experimentally confirm this inversion at both sides (Fig. 4c, d). The breakpoints of the inversion in CBS8639 are located in the intergenic regions between *KB390G02120* (annotated as “hypothetical protein” in *S. cerevisiae*) and *KB390G02130* (annotated as *CYT2* in *S. cerevisiae*) on one flank and *KB390G02820* (annotated as *AQY1* in *S. cerevisiae*) and *KB390G02810* (annotated as *YKL162C* in *S. cerevisiae*) on the other flank. In NRRL Y-27205, the up-stream region of the breakpoint, including where the F1 primer has been placed, is homologous to the other strains with the exception of the loss of the *KB390G02120* gene and the insertion at this locus of a different gene, *NR270G02130*. The downstream breakpoint is in the same inter-genic region as CBS 8639. While *KB390G02120* is also an annotated gene in *K. exigua, NR270G2130*, is part of the set of genes with no 1:1 ortholog after functional analysis, and no Alphafold prediction.

### Heterozygosity and sequence divergence among *K. bulderi* strains

To assess the overall genetic divergence within and between the three *K. bulderi* strains, we carried out pair-wise comparisons between the consensus genomes of the three strains (i.e. primary assemblies) and the total sequence reads of each strain (Supplementary Table 11). The data shows that NRRL Y-27205 genome is the most diverse compared to the other *K. bulderi* strains (i.e. 364.1 K intra-genomic variants vs 362.1 K and 347.9 K for CBS 8639 and CBS 8638, respectively), and also the more distant from the other two strains. *K. bulderi* shows a lower level of heterozygosity compared to *Saccharomyces cerevisiae*^37^, but a higher level of heterozygosity compared with other yeast, such as *Kluyveromyces marxianus*^38^. The complete breakdown for bi-allelic and multi-allelic sites are shown in Supplementary Table 12.

To assess the divergence between alleles, we compared all the SNPs located within conserved CDS (i.e. CDS shared by all strains). We identified ca. 14.1 K SNP sites that are in common with all three strains. Out of these, ca. 12.3 K share the same consensus and the same alternative SNPs, and only 1.8 K have different variants in the three strains. CBS 8639 and CBS 8638, which are more closely related, share ca. 68.4 K SNP sites, out of which 1.2 K have different variants; while CBS 8639 and NRRL Y-27205 share ca. 19.8 K SNPs sites of which 1.8 K have different variants.

Overall, the genetic divergence between CBS 8638 and CBS 8639 is lower than that observed between NRRL Y-27205 and CBS 8639. NRRL Y-27205 also shows the biggest phenotypic differences under the stress conditions tested (Supplementary Fig. 1–3).

We checked the number of genes that were homozygous (the two alleles are identical) in each strain and found that ca. 13% of genes are homozygous for CBS 8638 and CBS 8639 and ca. 18% in NRRL Y-27205 (Supplementary Data 4). Within these homozygous sets there is at least a three-fold enrichment of *K. bulderi* specific genes compared to the expected proportion when considering the whole genome data (*p* value < 0.00001, chi-square test). Furthermore, 234 genes are homozygous in all three strains. In this common set, there is also an over-representation (8.5 fold) of *K. bulderi* specific genes (*p* < 0.00001, chi-square test). The highest number of genes in homozygous regions are located in chromosome VII and VIII (Supplementary Data 4). Heterozygosity is often thought to be important for the initial adaptation to new habitats^39–41^. Species that have become adapted to highly selective environments tend subsequently to retain favorable alleles exhibiting homozygous regions within the genomes^42–46^.

### Comparison of *K. bulderi* vs other *Kazachstania* genomes

**Fig. 5:**
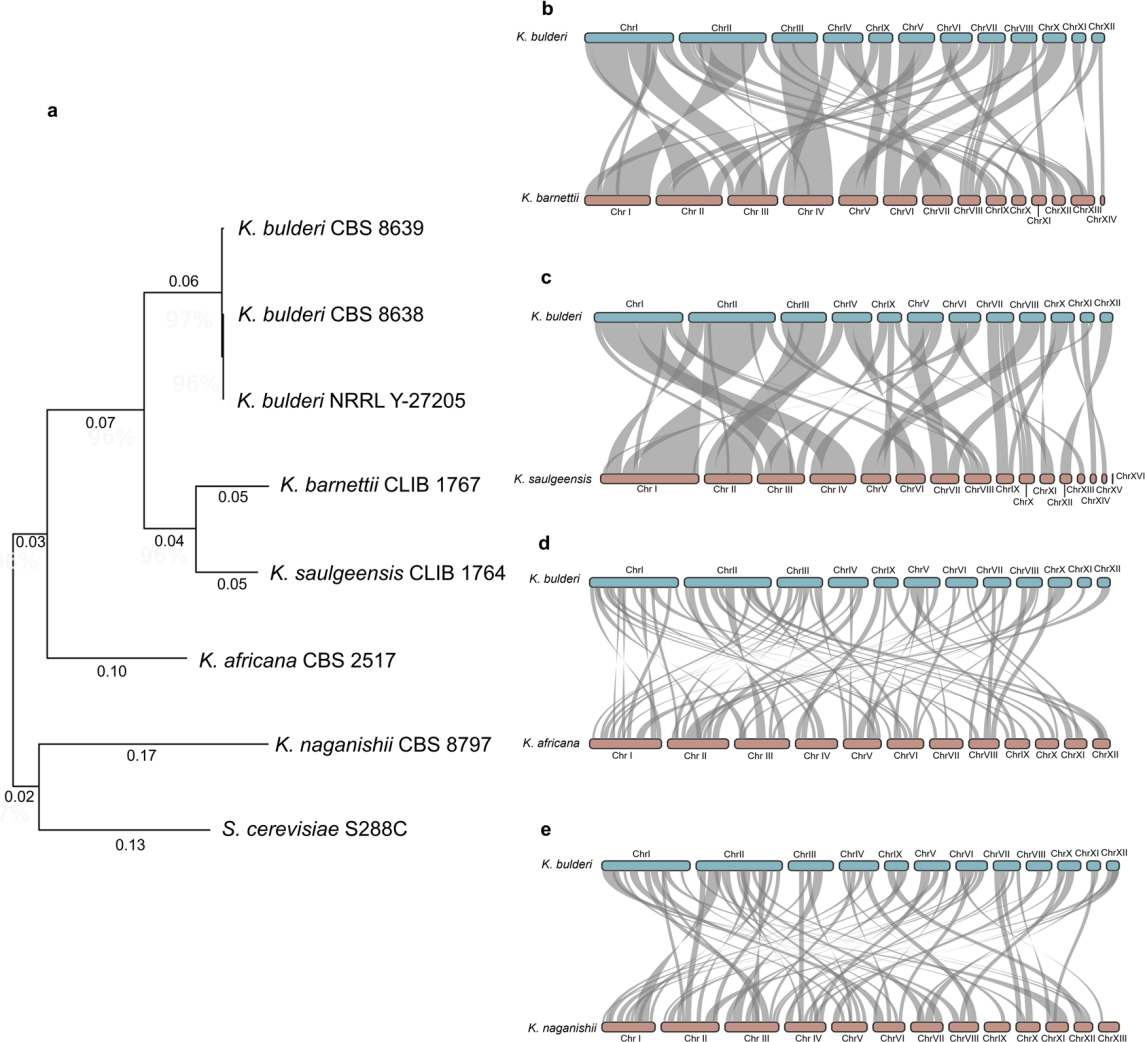
Phylogenetic relationships and synteny between *K. bulderi* and other *Kazachstania* species. **a** Phylogenetic tree reconstructed using Neighbor-Join and BioNJ algorithms of the combined sequences of 30 genes, depicting the relationship of *K. bulderi* strains with closely related *Kazachstania* that have been fully assembled. The tree is drawn to scale, with branch lengths measured in the number of substitutions per site. Synteny blocks between the *K. bulderi* CBS 8639 genome (light blue) and other *Kazachstania* species, including *Kazachstania barnettii* (**b**), *Kazachstania saulgeensis* (**c**), *Kazachstan africana* (**d**), and *Kazachstania naganishii* (**e**). The synteny blocks are represented by the gray areas between the CBS 8639 query genome and the other *Kazachstania* sp. genome.

The phylogenetic relationships between species of *Kazachstania* remain poorly understood, largely due to the lack of complete and well-assembled genomes. Previous studies have relied on phylogenetic trees based on D1/D2 domains of the LSU rDNA and ITS regions^15,47,48^ which have limited resolution. Four fully assembled genomes belonging to different *Kazachstania species* are currently publicly available (10–12) and were used together with our sequenced genomes of *K. bulderi* strains to draw the phylogenetic relationships and synteny between these species. We generated an updated tree using 30 conserved genes (Supplementary Table 13) and carried out a synteny analysis between *K. bulderi* CBS 8639 and the other *Kazachstania* species (Fig. 5b–e). Our results indicate that low pH tolerant species, *K. bulderi, K. barnettii*, and *K. saulgeensis*, are phylogenetically more closely related (Fig. 5a) and show higher synteny (Fig. 5b, c). In fact, *K. barnettii*, and *K. saulgeensis* show 16 and 15 synteny blocks larger than 100 Kb in common with *K. bulderi*, respectively (Supplementary Fig. 13a, b). Moreover, more than 90% of chromosome III maintains the same synteny and gene order. In contrast*, K. africana* and *K. naganishii*, which were isolated from soil^49^ and decayed leaves^50^, respectively, are more distant on the phylogenetic tree (Fig. 5a) and also showed the lowest level of synteny with *K. bulderi* (Fig. 5d, e) and smaller size of synteny blocks (Supplementary Fig. 13c, d). For additional details on the synteny blocks, refer to Supplementary Data 5 and Supplementary Figure 13.

### Inspection of mating type loci

A yeast diploid strain usually has a **MATa** and a *MATα* locus, however some diploid strains can be homozygous in the *MAT* locus and unable to sporulate^51^. *HML* and *HMR* are copies of the *MATα* and *MAT***a**, respectively, and, in homothallic strains, are used to repair the *MAT* locus during mating type switching initiated by the product of the HO endonuclease gene. While *Saccharomycetaceae* species are typically homothallic, there have been reports of transition to heterothallism (unable to perform mating type switching) in certain *Kazachstania* genera, such as *Kazachstania yakushimaensis* and *Kazachstania transvaalensis*^52^.

For the *K. bulderi* strains, the mapping of the *MAT* locus was carried out using *Saccharomyces cerevisiae*, *Kazachstania naganishii*, *Kazachstania saulgeensis,* and *Kazachstania barnettii* genomes as reference. Our analysis revealed that the *HML* and *MAT* loci are located on chromosome V in all three *K. bulderi* strains. Through structural and functional annotation, we identified 1:1 orthologs for the *MATα1* and *MATα2* genes in both the alternative and principal assemblies. By directly mapping the reads at the *MAT* locus we were also able to identify a region of the *MAT*a1 gene (Supplementary Fig. 14), and three truncated *HMR* loci, specifically *HMR1*a on chr IV, *HMR2*a on chr I, and *HMR3*a on chr XI. These data suggest that these strains are heterozygous in the *MAT* locus.

**Fig. 6:**
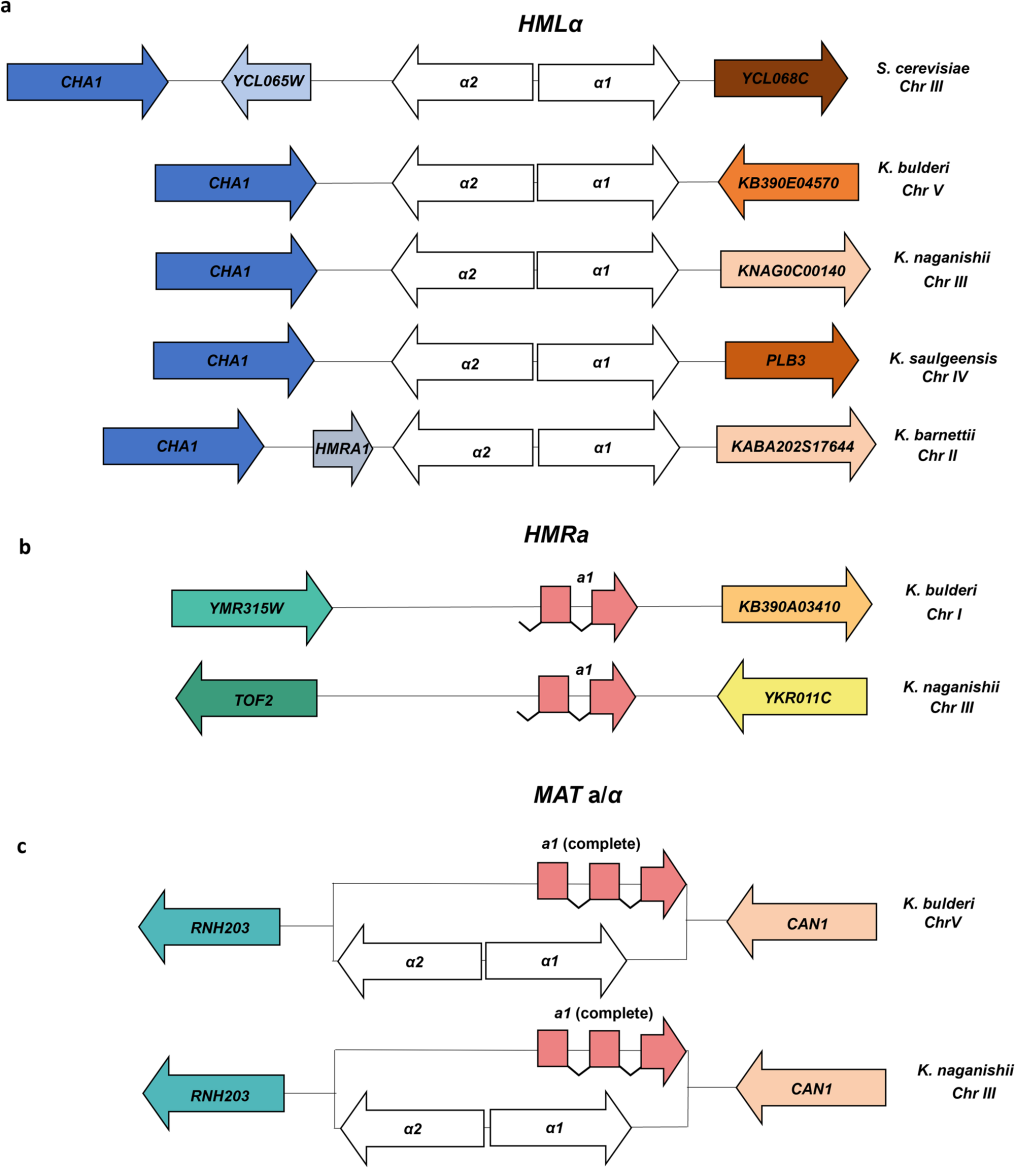
Comparative landscape of the mating type loci in *K. bulderi* and other yeast species. **a** Schematic organization of the *HML* locus in *K. bulderi, S. cerevisiae, K. naganishii, K. saulgeensis*, and *K. barnettii* strains. **b**, **c** Comparative schematic depiction of the *HMR* and *MAT* loci between *K. bulderi* with *K. naganishii*. The central blocks feature the *HML* type genes, including *alpha*1 and *alpha*2 (depicted in white), along with the *HMR*a1 gene highlighted in pink. Surrounding colored blocks correspond to adjacent flanking genes. Conserved regions marked by blocks of the same color across species denote shared syntenic genes.

The *HML* locus, including the neighboring genes (i.e. *CHA1*) on the X region (Fig. 6a), is broadly similar to that of *Kazachstania saulgeensis* and *Kazachstania naganishii*^16^. On the Z region of *HML* locus the neighboring genes differ in *K. bulderi* compared with the other *Kazachstania* species. Such organization of the *MAT* locus is likely due to a series of deletions following the whole genome duplication^17^. The *HMR* locus is conserved between *K. barnettii* and *K. saulgeensis*,^16^, but lost in *K. africana*^17^. Moreover, chromosomal rearrangements at the *MAT* locus are common in several *Kazachstania* species. For instance, *K. barnettii* shows an inversion between the *HMR* and *MAT* locus^16^, whereas in *K. africana* a translocation has caused the loss of the *HML* and *HMR* silent cassettes^17^. The arrangement of *HMR* locus in *K. bulderi* seems to be similar of that one of *K. naganishii* (Fig. 6b), where the copy of the a1 gene is truncated at the 5’ end (lacking a promoter or start codon). During the mating-type switching from *MATα* to *MAT*a the 3′ end of the a1 gene (exon 3) is inserted beside the 5′ end of the gene at the *MAT* locus (exons 1 and 2) to make a full-length and functional a1 gene (Fig. 6c)^53^. This organization allows *HMR* in *K. bulderi* to be effectively silenced without the requirement of being in a sub-telomeric region, as it only contains exon 2 and 3^52,53^. The HO endonuclease gene is present on chr I in all *K. bulderi* strains, suggesting that these strains are homothallic^52^.

### Mapping of the mtDNA in *K. bulderi*

**Fig. 7:**
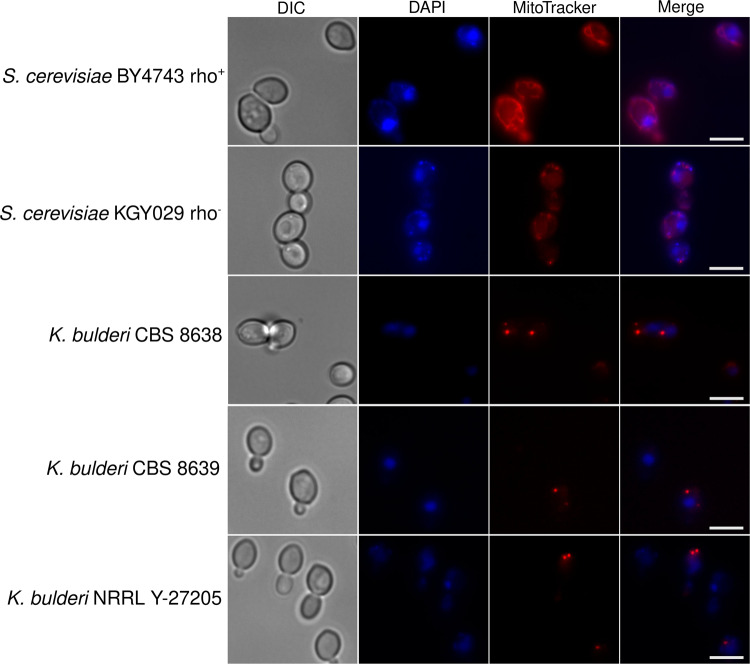
Mitochondrial DNA visualization in *K. bulderi* strains. DAPI and MitoTracker staining of *K. bulderi* CBS 8638, CBS 8639 and NRRL Y-27205 strains along with *S. cerevisiae* BY4743 [rho^+^] and KGY029 [rho^−^]. The columns represent the cells observed at differential interphase contrast (DIC), and the signal from DAPI (blue), MitoTracker (red) and merged DAPI and MitoTracker. Scale bars represent 5 µm.

Mitochondria play a crucial role in the processes that drive understanding of phylogenetic relationships and mechanisms of evolutionary adaptation^54–57^. We could not detect mitochondrial DNA using PacBio sequencing, suggesting that these strains may be deficient of mitochondrial DNA. This was further evidenced by the fact that none of the strains grew on glycerol as a sole carbon source (Supplementary Fig. 15a). We used DAPI stain to visualize the DNA content (nuclear and mitochondrial, the latter typically observed in the periphery of the cell) in the three strains. In addition, rho^+^ and rho^-^ *S. cerevisiae* control strains were used, which have functional mitochondrial DNA and damaged mitochondrial DNA, respectively^58^. Fluorescent spots corresponding to mitochondrial DNA (mtDNA) were observed in the *S. cerevisiae* rho^+^ and rho^-^ strains, indicating the presence of mtDNA in these strains. However, in the case of *K. bulderi* CBS 8638 and CBS 8639 strains, only nuclear DNA was observed, further validating that these strains are likely to be rho^0^, characterized by the absence of entire mitochondrial DNA (Supplementary Fig 15b). Alternatively, the staining of the mtDNA of NRRL Y-27205 was similar of the control rho^−^ and contained a mixed population of cells displaying either a rho^−^ or rho^0^ phenotype (Supplementary Fig. 15b). The cells were also stained with both DAPI and MitoTracker Red CMXRos, a specific dye for mitochondria, to confirm the lack of DNA at the mitochondria sites in the *K. bulderi* strains (Fig. 7). Moreover, in contrast to *S. cerevisiae* BY4743 (rho^+^), where mitochondria form a tubular network, all three *K. bulderi* strains exhibited punctuated mitochondria more similar to those observed in the *S. cerevisiae* KGY029 rho^−^ with damaged mtDNA (Fig. 7).

To further verify the presence/absence of mitochondrial DNA in these strains, we re-sequenced them using nanopore sequencing. We mapped the nanopore reads versus the mtDNA of *Kazachstania servazzii* of the size of 30,782 bp^21^. For CBS 8639 only one read partially mapped to an intergenic region of 200 bp in the mtDNA, while no reads of CBS 8638 mapped to any portion of the *Kazachstania servazzii* mtDNA, confirming the rho^0^ nature of the strains.

For the NRRL Y-27205 strain we found some evidence of mtDNA, since approximately 2,725 reads mapped to a region of 5,100 bp of the mtDNA from *Kazachstania servazzii* that includes three genes, namely *COX1, ATP8, ATP6*. We therefore propose to classify NRRL Y-27205 as rho^−^. Due to the low coverage in the mapped region and limited read length, no attempts were made to assemble these reads.

## Conclusions

We showed that *K. bulderi* strains are able to grow in the presence of high concentration of organic acids and show resistance to cationic drugs. A high-quality genome assembly of three *K. bulderi* strains was achieved through *i*. a comparison of algorithms for genome assembly and annotation, *ii*. manual curation and *iii*. experimental validation; and a fully annotated reference genome for this species was constructed. Moreover, we distinguished and validated chromosomal rearrangements in the *K. bulderi* strains studied in this work, that could contribute to their phenotypic variation. Extensive functional annotation revealed potential genus-specific and species-specific genes that might have evolved under the highly selective pressure of maize silage. We showed that the *K. bulderi* strains are overall closely related to *K. barnetii* and *K. saulgeensis*, recapitulating the phylogenetic tree, and the organization of the *MAT* cassette is similar to *K. naganishii*. *K. bulderi* strains have lost the mitochondrial DNA and are unable to grow on non-fermentable sources.

The current assemblies establish a basis for further studying the phylogeny of the *Kazachstania* genus, which can be used to investigate genomic events associated with yeast domestication and species radiation. Moreover, bespoke molecular tools can now easily be developed for biotechnological purposes, given such strains can optimally grow at low pH and tolerate high concentrations of organic acids.

## Methods

### Strains and phenotypic analysis

Three *K. bulderi* strains CBS 8638, CBS 8639, and NRRL Y-27205 and five *S. cerevisiae* strains 96.2, BY4743, BY4741, NCYC 505, and KGY029 were used in this study. A detailed description of the strains is listed in Supplementary Table 14.

For liquid fitness assays, *K. bulderi* and *S. cerevisiae* strains were grown at 30 °C overnight on YPD and then diluted to an OD_600_ of 0.1. Growth measurements at OD_595_ were recorded in every condition using a FLUOstar OPTIMA Microplate Reader (BMG). The OD_595_ measurements were taken by the microplate reader at 25 °C for 72 h at intervals of 5 min and with 1 min linear shaking before every read. Three technical and three biological samples were used for each *K. bulderi* and *S. cerevisiae* strains. For the lactic acid and the formic acid media, the pH was kept constant at 2.5 and 3, respectively, independently of the acid concentration. To adjust the pH either 10 M of NaOH or 1 M of H_3_PO_4_ was used. For determination of biomass, 100 mL flask fermentations were cultured for 6 days. Culture dry weights were determined by collecting the cells in a 0.45 µm-pore filters and oven dried at 100 °C overnight.

Graphs were generated by GraphPad Prism, version 9.0 and growth parameters were calculated using the “Growthcurver” R package^59^. For spot test assays on solid media, cultures were grown overnight at 30 °C and number of cells were normalized to an OD_600_ of 4 before being serially diluted 1:10 and spotted onto YPD agar plates containing 100 µg/mL, 150 µg/mL, 200 µg/mL and 300 µg/mL of hygromycin B (Invitrogen); 5 µg/mL, 10 µg/mL and 25 µg/mL of phleomycin (InvivoGen); 10 µg/mL and 15 µg/mL of Nourseothricin (Stratech Scientific Ltd), 100 µg/mL and 200 µg/mL G 418 (Sigma-Aldrich) and YP + 2% glycerol.

### DNA extraction and library preparation for long read next generation sequencing

For PacBio sequencing, the DNA was extracted from samples using the cetyl trimethyl ammonium bromide (CTAB) method^60^. For Nanopore sequencing, we used either the CTAB or the NucleoBond High Molecular Weight DNA Kit (Macherey-Nagel) following the manufacturer’s instructions. For the CTAB method, 1 mL of overnight culture was added to 50 mg of acid washed glass beads 425–600 µm (Sigma). 1 mL of CTAB extraction buffer was then added, and after incubation at 65 °C. RNAse A treatment was conducted by adding 2 µL of RNAse A 100 µg/mL (Qiagen) and incubating the samples at 37 °C for 15 min. After purification with phenol:chloroform:isoamyl alcohol (25:24:1) the DNA was eluted in 50 L of ultrapure distilled water (Invitrogen) and stored at 4 °C. The DNA quality was assessed using the NanoDrop LiTE Spectrophotometer (Thermofisher Scientific) to be within the quality specification range required by the PacBio and Oxford Nanopore protocols.

The genomic DNA was adjusted to 10 ng/µL in 150 µL and sheared to ~10 kilobase fragments using g-TUBES (Covaris) following the manufacturer’s instructions. The quality and size of DNA fragments were verified using Fragment Analyzer (Advanced Analytical Technologies) following the DNF-90 protocol. Samples were prepared for sequencing following the Express Template Prep Kit 2.0 protocol (Pacific Biosciences), with multiplexing using the Barcoded Overhang Adapter kit 8A (Pacific Biosciences). DNA libraries were sequenced using the SMRT Cell 1 M chips on the Pacific Biosciences Sequel system with 10 h data acquisition time. For Nanopore sequencing, 1 µg of DNA samples (not sheared) were prepared for sequencing using either the SQK-LSK109 Ligation sequencing kit or the SQK-RBK004 Rapid Barcoding Kit^61^ followed by the Flongle sequencing expansion kit FLO-FLG001 (both Oxford Nanopore), following the manufacturer’s instructions. Each strain was sequenced using a MinION Flongle flow cell with 24 h data acquisition time. Higher number of coverage and reads were obtained using the SQK-LSK109 kit, so we only reported the data and analysis using this protocol.

### Genome assembly

PacBio sequencing data was processed to generate circular consensus sequencing (CCS, or HiFi) reads using the CCS application in SMRT Link 8.0 software package with three passes, considered to generate a minimum Q20 accuracy and to mitigate homopolymer frameshifts^62–64^. The CCS read length ranged from 10 to 50,000. CCS reads were assembled using the PacBio assembler algorithm’s Improved Phased Assembly (IPA v1.8.0) method (available at https://github.com/PacificBiosciences/pbipa.git) and the HiFiasm assembly tool^65^ with default settings. Both genome assembler’s output consists of one primary contig and one alternate haplotig files that were converted to FASTA format.

### Curation and polishing of the definitive genome assembly

Final adjustments of the selected genome assembly were made manually based on the assembly graph, read coverage and distribution and experimental validations. By using de novo assemblies of the three *K. bulderi* strains we were able to build a reference genome by i. visualizing the assembly alignment by Bandage V0.8.1^66^; ii. using contiguous edges between split contigs (nodes) for each *K. bulderi* strain identified using the other two strains as references (Supplementary Note 1); iii. extracting candidates’ sequences for long PCR validations in order to resolve incomplete/split contigs (Supplementary Note 2, 3); iv. identifying repetitive regions located at the ends of contigs that are likely to represent telomeric regions; v. mapping the HiFi reads by minimap2 v2.24^67,68^ of each strain against each de novo assembly to identify linear read coverage and resolve potential contigs translocated or misplaced by the assembly tool used. The alignments were visualized by Ribbon interactive online visualization tool^69^ available http://genomeribbon.com. Finally, assembled sequences were visualized and compared against the final assembly using the BLAST v2.12.0^70^, to obtain the final polished assembly. Genome assembly statistics about quality and contiguity were assessed using QUAST v5.0.114^71^ at both contig and chromosomal level. To assess completeness we used BUSCO v5.3.2^31^, based on T = the lineage dataset: saccharomycetes_odb10 (number of genomes: 76, number of BUSCOs: 2137) in both polished and unpolished assemblies. Circos plot of the curated final *K. bulderi* genome assembly was generated with Circa (http://omgenomics.com/circa).

### Structural and functional annotations

Gene annotation and gene prediction was achieved using The Yeast Genome Annotation Pipeline (YGAP)^29^ and AUGUSTUS v3.4.0^28^ for all the de novo *K. bulderi* genome assemblies. AUGUSTUS predicts genes from start to stop codon using a hidden Markov model while YGAP uses homology and synteny information from other yeast species present in the Yeast Gene Order Browser database to predict the gene structure (based on the hypothesis that the genes intron/exon structure is conserved through evolution). The HybridMine tool v4.0^30^, initially developed for functional annotation at gene level was modified to work at protein level and used to identify one-to-one orthologs between the *K. bulderi* strains and *S. cerevisie*, *Y. lipolytica*, *S. pombe*, *C. albicans*, *C. glabrata, K. exigua*, *K. marxianus*, *K. lactis,* and *K. barnetti*, respectively. HybridMine was also used to identify groups of homologs within each *K. bulderi* strain genome and to identify the genes shared by the three *K. bulderi* strains. Visualization of the proteins shared between the different species was carried out using the “ComplexUpset” and “ggplot2” R packages. Protein structure prediction for 281, 245, and 281 non-annotated proteins predicted by YGAP in *K. bulderi* CBS 8638, CBS 8639 and NRRL-Y27205, respectively, has been carried out using AlphaFold v2.1.1^32^. Functional annotation from the predicted 3D structures was then achieved using DeepFRI v1.0.0^33^.

### Comparative genome analysis

For analysis of intra and interspecific variation pbmm2 v1.10.0 (available at https://github.com/PacificBiosciences/pbmm2), a SMRT C++ wrapper for minimap2’s C API, was used to index the reference genomes and align the sequencing reads to the references. SAMtools v1.10 suite has been used to process the sequence alignment files^72^. BAM files were sorted and indexed for SNP calling using *samtools sort* and *samtools index*, respectively. DeepVariant v1.5.0^73^ was used for variant calling. BCFTools v1.10.2 was used for manipulating VCFs and BCFs. The variant analysis was performed inside the CDS regions to calculate heterozygous sites within each genome using an in-house python 3 script. The same variant combination and different variant combination between the heterozygous site was also detected. Gene analysis for homozygosity was carried out to using an in-house python3 script. Chi-square statistical test was performed to analyze the representation of *K. bulderi* specific genes among the genes within homozygous regions in each strain using R.

The dot plots were generated using D-GENIES^74^, an online tool available at http://dgenies.toulouse.inra.fr/. As all *K. bulderi* genome assemblies were constructed de novo, alignments were also made to the unorder and initial scaffolds using MUMmer v3.0^75^ to confirm chromosomal orientation. To determine the level of synteny and visualize the synteny blocks between *K. bulderi* and other *Kazachstania* species the ShinySyn application was used^76–78^. Evolutionary analysis was conducted in MEGA11 v11.0.11^79^. This analysis involved six species, including three *K. bulderi* strains, *K. barnettii*, *K. saulgeensis*, *K. africana*, *K. naganishii,* and *S. cerevisiae*, and 30 conserved gene sequences (a total of 59,290 nucleotides; Supplementary Table 12). The evolutionary history was inferred by using the Maximum Likelihood method and Tamura-Nei model^79^ on the concatenated alignment of the 30 conserved genes. The tree with the highest log likelihood (−233855.04) has been selected. Initial tree(s) for the heuristic search were obtained automatically by applying Neighbor-Join and BioNJ algorithms to a matrix of pairwise distances estimated using the Tamura-Nei model, and then selecting the topology with superior log likelihood value.

### PCR experimental validation and electrophoretic karyotype

The continuity of the contigs and the chromosomal rearrangements were validated by PCR. The PCR mixture composed 12.5 μL of 5X LongAmp Taq Reaction Buffer (NEB), 0.4uM of forward and reverse primers (Invitrogen), 2.5U of LongAmp Taq DNA Polymerase (NEB) and 1uL of gDNA. The mix was brought up to 25 μL, final volume, with ultrapure H_2_O. Cycling conditions were set up following the manufacturer’s protocol with the annealing temperature between 55 and 58 °C. 10 μL of PCR product was loaded on 1% (w/v) agarose gel electrophoresis in 1 × TAE buffer with a 5 μL/100 mL of SafeView nucleic acid stain. Samples were compared to 1 Kb hyper ladder (Bioline). Primer sequences used for long amplicons between contig edges and validation of chromosomal rearrangements are listed in Supplementary Tables 15–17. The electrophoretic karyotype was determined using pulsed-field gel electrophoresis (PFGE) with a CHEF-DR III system (Bio-Rad). For PFGE preparation, cells were grown in 50 mL of YPD for 72 h. Cells equivalent to OD600 = 5 were harvested and washed with a solution containing 0.5 mg/mL of lyticase (Sigma, L2524-50KU). These cells were mixed with an equal amount of 1.6% SeaKem LE agarose (Lonza) and pipetted into a 1.2 mm-thick plug mold (BioRad). After solidification (at 0–4 °C for 30 min), the plugs were incubated in a 100 mM EDTA solution that included 0.2% sodium deoxycholate, 1% sodium lauryl sarcosine, and 2 mg/mL of Proteinase K (Sigma) at 55 °C overnight. The plugs were rinsed with a solution containing 10 mM Tris-HCl pH 7.5 and 50 mM EDTA, and then stored at 4 °C (80). One-third of the plug was used for electrophoresis. The electrophoresis was run for a total of 30 h with a cooling temperature of 14 °C in two blocks. Initially for 15 h, 120° angle 4.5 V/cm switching time 60 s. The second block for 15 h, 100° angle, 4.5 V/cm and switching time 150 s.

### DAPI and MitoTracker staining and mapping of mtDNA

DNA analysis was performed by DAPI (4,6-diamidino-2-phenylindole; Sigma) staining. Yeast cells were harvested after overnight growth in YP + 2% glycerol and washed twice with PBS. Cells were resuspended in SD media w/o amino acids. DAPI and SDS were added to the culture at the final concentration of 1 µg/ml and 0.01%, respectively. The cells were incubated in the dark for 10 min at 30 °C. Mitochondria were visualized using Mitotracker Red CMXRos (Invitrogen), a red-fluorescent dye which stains mitochondria in live cells. Mitotracker Red was dissolved in DMSO to 2 mg/mL and added to the cells to a final concentration of 50 nM, cells were then incubated in the dark for 30 min at 30 °C.

Cells were observed with an Eclipse TE2000-U fluorescence inverted microscope (Nikon) fitted with a ×100 immersion objective. The images were captured using the Ocular Image Acquisition Software V2.0 (QImaging). The images were then processed and assembled with Image J^80,81^.

For Nanopore data, base calling was performed using Guppy v2.3.5 (Oxford Nanopore) and the reads from CBS 8638, CBS 8639, and NRRL Y-27205 were aligned against the sequence of mtDNA from *K*. *servazzii* using minimap2 v2.24. The aligned mitochondria reads were extracted and remapped against the *K. bulderi* genome assemblies to rule out mapping to previous annotated nuclear genes.

### Reporting summary

Further information on research design is available in the Nature Portfolio Reporting Summary linked to this article.

### Supplementary information

Supplementary Information

Description of Additional Supplementary Files

Supplementary Data 1

Supplementary Data 2

Supplementary Data 3

Supplementary Data 4

Supplementary Data 5

Reporting Summary

## Data Availability

The genome assemblies for strains CBS 8638, CBS 8639 and NRRL Y-27205 generated in this study have been deposited in the National Library of Medicine database (https://www.ncbi.nlm.nih.gov/) under the accession numbers SAMN32971551, SAMN32971552, SAMN32971553, respectively. The BioProject number is PRJNA929900. Scripts developed for the variant calling and the homozygosity/heterozygosity analysis are available in GitHub at https://github.com/Sookie-S/Variant_analysis_pacbio_data/tree/main/Scripts. Uncropped gel images corresponding to Fig. 4 and Supplementary Figs. 6–9 are included in Supplementary Figs. 16–18. Source data underlying Fig. 3 are included in Supplementary Data 2.
