# Supplementary material for: High quality de novo genome assembly of the non-conventional yeast Kazachstania bulderi describes a potential low pH production host for biorefineries
Source: Commun Biol. 2023 Sep 7;6:918. doi: 10.1038/s42003-023-05285-0 (PMC10484914; doi:10.1038/s42003-023-05285-0)
Supplement: Supplementary file 1 — Supplementary Information [file 42003_2023_5285_MOESM1_ESM.pdf]

Supplementary Information

Supplementary for: High quality *de novo* genome assembly of the non-conventional yeast, *Kazachstania bulderi* describes a potential low pH production host for biorefineries.

Laura N. Balarezo-Cisneros[1,†], Soukaina Timouma[1,†], Alistair Hanak[1], Andrew Currin[1], Fernando Valle[2], and Daniela Delneri[1,*]

[1] Manchester Institute of Biotechnology, University of Manchester, Manchester, UK
[2] BP Biosciences Center, San Diego, California, USA
[†]These two authors contributed equally
[*]corresponding author: d.delneri@manchester.ac.uk

Additional Information is available for this paper:

Supplementary Tables 1-17 (available in this file)

Supplementary Figures 1-18 (available in this file)

Supplementary Notes (1-3) (available in this file)

Supplementary Data 1-5 (available as separate Excel files)

Supplementary Tables.

Supplementary Table 1. Growth parameters calculated at different concentration of lactic acid and formic acid. The table shows the mean and standard deviation of the growth rate, doubling time and area under the curve across three biological replicates.

Strains	Conditions	Growth rate *r* [h^{-1}]	Doubling Time t_{DT} [h]	Area under the curve (AUC)
CBS 8638	SD+ 50g/L Lactic acid	0.19 ± 0.00	3.61 ± 0.05	108.32 ± 3.10
CBS 8639		0.16 ± 0.00	4.38 ± 0.08	99.32 ± 5.37
NRRL Y-27205		0.14 ± 0.02	5.10 ± 0.66	110.14 ± 5.18
BY4743		0.03 ± 0.00	22.13 ± 2.73	8.54 ± 0.72
CBS 8638	SD+ 60g/L Lactic acid	0.22 ± 0.01	3.09 ± 0.08	113.48 ± 2.81
CBS 8639		0.15 ± 0.01	4.73 ± 0.18	104.56 ± 5.25
NRRL Y-27205		0.15 ± 0.01	4.75 ± 0.19	98.42 ± 10.12
BY4743 *		0.07 ± 0.01	10.84 ± 1.80	2.68 ± 0.48
CBS 8638	SD+ 75g/L Lactic acid	0.32 ± 0.01	2.14 ± 0.09	79.13 ± 1.47
CBS 8639		0.30 ± 0.02	2.35 ± 0.17	72.99 ± 3.54
NRRL Y-27205		0.20 ± 0.00	3.43 ± 0.05	67.18 ± 2.10
CBS 8638	SD+ 80g/L Lactic acid	0.26 ± 0.01	2.64 ± 0.07	77.45 ± 2.30
CBS 8639		0.24 ± 0.01	2.88 ± 0.13	71.21 ± 2.41
NRRL Y-27205		0.06 ± 0.01	12.12 ± 2.11	25.59 ± 8.47
CBS 8638	SD+ 85g/L Lactic acid	0.19 ± 0.03	3.62 ± 0.50	106.67 ± 3.88
CBS 8639		0.08 ± 0.00	8.23 ± 0.32	90.52 ± 5.44
NRRL Y-27205		0.08 ± 0.01	8.53 ± 1.31	12.62 ± 1.52
CBS 8638	SD+ 25mM formic acid	0.50 ± 0.01	1.40 ± 0.02	65.50 ± 2.43
CBS 8639		0.47 ± 0.01	1.48 ± 0.04	64.22 ± 3.29
NRRL Y-27205		0.43 ± 0.02	1.60 ± 0.06	62.30 ± 4.04

* BY4743 does not grow on lactic acid ≥ 50g/L or/and formic acid ≥ 25mM

Supplementary Table 2. Values of biomass yield (*Y*) on glucose *K. bulderi* strains CBS 8638, CBS 8639 and NRRL Y-27205 and *S. cerevisiae* NCYC 505 and BY4743 strains grown in SD at pH 5.5, and 2.5, SD + 75 g/L lactic acid and SD + 25mM formic acid. *Y* is given in grams of biomass per gram of glucose utilized.

Condition	Strain	*Y*g/g
SD at pH 5.5	Kb CBS8638	0.063
	Kb CBS8639	0.057
	Kb NRRL Y-27205	0.067
	Sc NCYC 505	0.097
	Sc BY4743	0.077
SD at 2.5	Kb CBS8638	0.07
	Kb CBS8639	0.067
	Kb NRRL Y-27205	0.06
	Sc NCYC 505	0.04
	Sc BY4743	0.053
SD + 75 g/L lactic acid at pH 2.5	Kb CBS8638	0.037
	Kb CBS8639	0.027
	Kb NRRL Y-27205	0.003
	Sc NCYC 505	NG
	Sc BY4743	NG
SD + 25mM formic acid at pH 3.0	Kb CBS8638	0.033
	Kb CBS8639	0.01
	Kb NRRL Y-27205	0.027
	Sc NCYC 505	NG
	Sc BY4743	NG

NG= no growth

Supplementary Table 3. Assembly statistics of raw PacBio *K. bulderi de novo* genome assemblies by different approaches IPA and Hifiasm

		IPA			Hifiasm	
		CBS 8638	*CBS 8639*	*NRRL Y-27205*	*CBS 8639*	*CBS 8638*
Primary contig assembly	Size (Mb)	14.47	14.03	14.32	17.17	19.96
	N° Contigs	17	14	15	40	26
	Largest contig (bp)	2734419	2786749	2758527	3868179	3273188
	N50 (Mb)	1.37	1.12	1.10	1.51	2.44
	N75 (Mb)	0.71	0.86	0.73	1.14	1.63
	L50	4	4	4	3	4
	L75	8	7	8	6	6
	GC (%)	33.12	33.11	33.16	33.45	33.2
Alternate contigs	Size (Mb)	13.37	14.41	16.11	10.81	7.66
	N° Contigs	85	108	172	38	33
	Largest contig (bp)	1622056	2499223	1891183	1116028	1309555
	N50 (Mb)	0.92	1.13	0.95	0.67	0.69
	N75 (Mb)	0.42	0.54	0.49	0.52	0.39
	L50	6	5	7	7	4
	L75	11	9	12	12	8
	GC (%)	33.11	33.01	33.05	33.15	33.23

Supplementary Table 4. Summary statistics of the PacBio reads mapped to the IPA and Hifiams *K.bulderi* assemblies

Feature	IPA			Hifiasm	
	CBS 8638	CBS 8639	NRRL Y-27205	CBS 8638	CBS 8639
Mean read length	6504.7	7083.2	7540.8	6,191.70	6,709.20
Median read length	6289	7042	7538	6,007.00	6,817.00
Number of reads	131888	125890	159349	145,295.00	130,073.00
Read length N50	8065	8201	8927	7,950.00	8,166.00
Total bases	857894356	891703780	1201624508	899,629,549.00	872,685,868.00
Coverage X	59.30	63.54	83.92	45.07	50.84

Supplementary Table 5. Number of proteins functional annotated in common between the principal and alternative assembly for CBS 868, CBS 86339 and NRRL Y-27205

	CBS 8638 principal	CBS 8638 alternative	CBS 8639 principal	CBS 8639 alternative	NRRLY-27205 principal	NRRLY-27205 alternative
Number of genes annotated	5877	3329	5759	4252	5769	2804
1:1 orthologs between principal and alternative	3129		3928		2574	

Supplementary Table 6. Comparison of the initial number of contigs between the primary assemblies for the three *K. bulderi* strains. Similar contigs were assign the same number followed by letters a, b, c to show connections.

Strain	Name Original assembly IPA	Initial nomenclature
CBS 8639	ctg.000000F	LB Chr I
	ctg.000001F	LB Chr II
	ctg.000006F	LB Chr III
	ctg.000008F	LB Chr IV
	ctg.000003F	LB Chr V
	ctg.000011F	LB Chr VI
	ctg.000005F	LB Chr VII
	ctg.000016F	LB Chr VIII a
	ctg.000023F	LB Chr VIII b
	ctg.000009F	LB Chr IX
	ctg.000015F	LB Chr X
	ctg.000020F	LB Chr XI
	ctg.000014F	LB Chr XII
	ctg.000022F	LB Chr XIII
CBS 8638	ctg.000001F	LB Chr I a
	ctg.000002F	LB Chr I b
	ctg.000015F	LB Chr I c
	ctg.000000F	LB Chr II
	ctg.000005F	LB Chr III
	ctg.000004F	LB Chr IV
	ctg.000008F	LB Chr V
	ctg.000012F	LB Chr VI
	ctg.000027F	LB Chr VII a
	ctg.000026F	LB Chr VII b
	ctg.000006F	LB Chr VII c

	ctg.000014F	LB Chr VIII
	ctg.000007F	LB Chr IX
	ctg.000018F	LB Chr X a
	ctg.000017F	LB Chr X b
	ctg.000020F	LB Chr XI
	ctg.000019F	LB Chr XII a
	ctg.000015F	LB Chr XII b
	ctg.000004F	LB Chr XIII
NRRL Y-27205	ctg.000000F	LB Chr I
	ctg.000001F	LB Chr II
	ctg.000010F	LB Chr III a
	ctg.000020F	LB Chr III b
	ctg.000008F	LB Chr IV a
	ctg.000024F	LB Chr IV b
	ctg.000003F	LB Chr V
	ctg.000009F	LB Chr VI
	ctg.000005F	LB Chr VII
	ctg.000013F	LB Chr VIII
	ctg.000002F	LB Chr IX
	ctg.000015F	LB Chr X
	ctg.000017F	LB Chr XI
	ctg.000018F	LB Chr XII
	ctg.000023F	LB Chr XIII

Supplementary Table 7. |Comparison of the BUSCO statistics on the three *K. bulderi* strains for the initial and polished assemblies.

BUSCO score (%)			
	CBS 8638	CBS 8639	NRRL Y-27205
Initial assembly			
Complete	98.90%	97.60%	97.30%
Fragmented	0.30%	0.30%	0.30%
Missing	0.80%	2.10%	2.40%
Polished assembly			
Complete	99.00%	99.10%	98.70%
Fragmented	0.20%	0.30%	0.20%
Missing	0.80%	0.60%	1.10%

Supplementary Table 8. Number of genomic elements annotated on *K. bulderi* strains per chromosome. *Chromosome VIII contains rRNA repetitions.

	Chromosome	Size (Mb)	CDS	tRNAs	Ty
CBS 8639	Chr I	2.79	1093	40	8
	Chr II	2.72	1091	30	4
	Chr III	1.42	538	24	3
	Chr IV	1.24	405	19	4
	Chr V	1.12	454	13	2
	Chr VI	0.99	380	15	2
	Chr VII	0.86	341	9	0
	Chr VIII*	0.80	297	12	4
	Chr IX	0.75	264	14	2
	Chr X	0.72	271	18	3
	Chr XI	0.43	145	7	0
	Chr XII	0.40	156	7	3
		Total	5435	208	35
CBS 8638	Chr I	2.77	1090	40	7
	Chr II	2.73	1091	30	4
	Chr III	1.37	527	24	3
	Chr IV	1.25	488	19	4
	Chr V	1.18	460	13	2
	Chr VI	0.94	377	15	3
	Chr VII	0.78	341	9	0
	LB Chr VIII*	0.80	294	12	5
	LB Chr IX	0.73	259	14	2
	LB Chr X	0.71	266	18	2
	Chr XI	0.43	152	7	1
	Chr XII	0.40	159	7	3
		Total	5504	208	36
NRRL Y-27205	Chr I	2.76	1083	39	6
	Chr II	2.72	1089	30	6
	Chr III	1.43	542	24	3
	Chr IV	1.22	482	19	3
	Chr V	1.23	473	13	2
	Chr VI	1.01	380	15	0
	Chr VII	0.90	346	9	0
	Chr VIII*	0.84	294	12	11
	Chr IX	0.73	261	14	2
	LB Chr X	0.65	250	18	3
	LB Chr XI	0.55	171	7	0
	LB Chr XII	0.40	154	7	1
		Total	5525	207	37

Supplementary Table 9. Number of *K. bulderi* functionally annotated proteins using genomes from different yeast species as references.

Yeast species	CBS 8638	CBS 8639	NRRL Y-27205
S. cerevisiae	4541	4543	4523
Y. lipolytica	3575	3591	3578
S. pombe	3058	3065	3062
C. albicans	3833	3838	3819
C. glabrata	4395	4404	4386
K. marxianus	4199	4211	4187
K. lactis	4296	4298	4276
K. exigua	5056	5069	5041
K. barnettii	5076	5086	5049

Supplementary Table 10. Location of *PDC*1 and *ZWF1* genes in CBS8638, CBS8639 and NRRL Y-27205 strains and their young paralogs.

Strain	Gene name	Systematic name	Chr	Position Start end	Visualisation	Chr size	Comment
CBS 8639	*PDC1*	KB390I00120	ChrIX	30319 32010		732771	beginning of chromosome * close to 30Kb telomeric region
	PDC1.2	KB390E04590	ChrV	1090658 1092349		1120440	end of chromosome * within 30Kb telomeric region
CBS 8638	*PDC1*	KB380I02680	ChrIX	696832 698523		732771	end of chromosome * close to 30Kb telomeric region
	PDC1.2	KB380E00220	ChrV	61798 63489		1177239	beginning of chromosome
NRRLY-27205	*PDC1*	NR270E00130	ChrV	27946 29637		1229362	beginning of chromosome * within 30Kb telomeric region
	PDC1.2	NR270E04740	ChrV	1200093 1201784		1229362	end of chromosome * within 30Kb telomeric region
	PDC1.1	NR270K01670	ChrXI	469171 470985		550191	end of chromosome * within 30Kb telomeric region

Strain	Gene name	Systematic name	Chr	Position Start end	Visualisation		Comment
CBS 8639	*ZWF1*	KB390A06340	ChrI	1605392 1606918		2786749	middle of chromosome
	ZWF1.1	KB390K01490	ChrXI	399322 400764		434096	near the end of chromosome * close to 30Kb telomeric region
	ZWF1.2	KB390A11040	ChrI	2757768 2759270		2786749	end of chromosome * within 30Kb telomeric region
CBS 8638	*ZWF1*	KB380A06280	ChrI	1587717 1589243		2767906	middle of chromosome
	ZWF1.1	KB380K01600	ChrXI	418110 419552		432092	end of chromosome * within 30Kb telomeric region
NRRLY-27205	*ZWF1*	NR270A04900	ChrI	1182534 1184060		2758527	middle of chromosome
	ZWF1.1	NR270K01570	ChrXI	425241 426683		550191	near the end of chromosome
	ZWF1.2	NR270A00160	ChrI	24904 26406		2758527	beginning of chromosome * within 30Kb telomeric region

Supplementary Table 11. Total number of genetic variants inside *K. bulderi* strains including single nucleotide polymorphisms (SNPs) and insertion/deletion variants (INDELs). Each reference genome was compared against the total sequence reads of each strain.

Reference	CBS 8638 reads			CBS 8639 reads			NRRL Y-27205 reads		
	SNPs	Indels	Total	SNPs	Indels	Total	SNPs	Indels	Total
CBS 8638	319.8K	28K	347.9K	332.4K	28.9K	361.4K	354.5K	30.9K	385.5K
CBS 8639	339.8K	29.9K	369.8K	333K	29K	362.1K	365.4K	32K	397.4K
NRRL Y-27205	360.2K	32.1K	392.4K	363.5K	32.1K	395.7K	334.7K	29.4K	364.1K

Supplementary Table 12. Number and breakdown of the type of SNPs and INDELs within *K. bulderi* strains**.**

Reference	CBS 8638 reads						
	Biallelic* insertion	Biallelic deletion	Biallelic SNP	Multiallelic* insertion	Multiallelic deletion	Multiallelic SNP	Multiallelic Complex**
CBS 8638	12.85K	15.09K	319.8K	19	13	5	49
CBS 8639	13.22K	16.60K	339.8K	22	27	17	74
NRRL Y-27205	14.69K	17.19K	360.2K	53	31	24	118

Reference	CBS 8639 reads						
	Biallelic insertion	Biallelic deletion	Biallelic SNP	Multiallelic insertion	Multiallelic deletion	Multiallelic SNP	Multiallelic Complex
CBS 8638	13.22K	15.62K	332.3K	33	29	6	72
CBS 8639	12.71K	16.27K	332.9K	27	13	0	55
NRRL Y-27205	14.66K	17.28K	363.4K	49	27	16	109

Reference	NRRL Y-27205 reads						
	Biallelic insertion	Biallelic deletion	Biallelic SNP	Multiallelic insertion	Multiallelic deletion	Multiallelic SNP	Multiallelic Complex
CBS 8638	14.12K	16.53K	354.5K	109	82	14	108
CBS 8639	14.12K	17.64K	365.3K	89	67	11	108
NRRL Y-27205	13.30K	15.93K	334.7K	77	43	3	70

*Biallelic variants are those where there is only one alternate allele, while multiallelic variants have more than one alternate allele. **Multiallelic Complex include those cases where there is a mixture of SNPs and indels for the same locus.

Supplementary Table 13. List of conserved genes used for the construction of the phylogenetic tree.

Gene Systematic Name	Gene Standard name	Protein name	Description
YGL123W	*RPS2*	40S ribosomal protein S2	Ribosomal Protein of the Small subunit
YHL015W	*RPS20*	40S ribosomal protein S20	Ribosomal Protein of the Small subunit
YGL189C	*RPS26A*	40S ribosomal protein S26-A	Ribosomal Protein of the Small subunit
YJR123W	*RPS5*	40S ribosomal protein S5	Ribosomal Protein of the Small subunit
YER102W	*RPS8B*	40S ribosomal protein S8-B	Ribosomal Protein of the Small subunit
YLR340W	*RPP0*	60S acidic ribosomal protein P0	Ribosomal Protein P0
YIL133C	*RPL16A*	60S ribosomal protein L16-A	Ribosomal Protein of the Large subunit
YOR063W	*RPL3*	60S ribosomal protein L3	Ribosomal Protein of the Large subunit
YGL030W	*RPL30*	60S ribosomal protein L30	Ribosomal Protein of the Large subunit
YPL143W	*RPL33A*	60S ribosomal protein L33-A	Ribosomal Protein of the Large subunit
YER091C	*MET6*	5-methyltetrahydropteroyltriglutamate--homocysteine methyltransferase	METhionine requiring
YNR016C	*ACC1*	Acetyl-CoA carboxylase	Acetyl-CoA Carboxylase
YPR145W	*ASN1*	Asparagine synthetase [glutamine-hydrolyzing] 1	ASparagiNe requiring
YMR205C	*PFK2*	ATP-dependent 6-phosphofructokinase subunit beta	PhosphoFructoKinase
YMR186W	*HSC82*	ATP-dependent molecular chaperone HSC82	
YJL050W	*MTR4*	ATP-dependent RNA helicase DOB1	Mrna TRansport
YPR010C	*RPA135*	DNA-directed RNA polymerase I subunit RPA135	RNA Polymerase A
YOR341W	*RPA190*	DNA-directed RNA polymerase I subunit RPA190	RNA Polymerase A
YER025W	*GCD11*	Eukaryotic translation initiation factor 2 subunit gamma	General Control Derepressed
YPL231W	*FAS2*	Fatty acid synthase subunit alpha	Fatty Acid Synthetase
YBR196C	*PGI1*	Glucose-6-phosphate isomerase	PhosphoGlucoIsomerase
YGL245W	*GUS1*	Glutamate--tRNA ligase, cytoplasmic	GlUtamyl-tRNA Synthetase

YGR192C	*TDH3*	Glyceraldehyde-3-phosphate dehydrogenase 3	Triose-phosphate DeHydrogenase
YMR116C	*ASC1*	Guanine nucleotide-binding protein subunit beta-like protein	Absence of growth Suppressor of Cyp1
YLR175W	*CBF5*	H/ACA ribonucleoprotein complex subunit CBF5	Centromere Binding Factor
YKL152C	*GPM1*	Phosphoglycerate mutase 1	Glycerate PhosphoMutase
YAL038W	*CDC19*	Pyruvate kinase 1	Cell Division Cycle
YLR180W	*SAM1*	S-adenosylmethionine synthase 1	S-AdenosylMethionine requiring
YPL082C	*MOT1*	TATA-binding protein-associated factor MOT1	Modifier of Transcription
YIL078W	*THS1*	Threonine--tRNA ligase, cytoplasmic	THreonyl tRNA Synthetase

Supplementary Table 14. Genotype and sources of yeast strains used in this study.

Strain		Genotype and auxotrophic markers	Source
S. cerevisiae	BY4741	*MATa his3Δ1 leu2Δ0 met15Δ0 ura3Δ0;* [rho^{+}]	Invitrogen
	BY4743	*MATa/α his3Δ1/his3Δ1 leu2Δ0/leu2Δ0 LYS2/lys2Δ0 met15Δ0/MET15 ura3Δ0/ura3Δ0;* [rho^{+}]	Invitrogen
	KGY029	*Mata leu2Δ0 leu2-3,112 ura3-52 his3::HindIII arg8::hisG ;* [rho^{-} *COX2*::*ARG8*m]	Cai's lab
	NCYC 505	prototroph	NCYC*<br>Brewer's top yeast, isolated from beer, Oranjeboom brewery, Rotterdam, Netherlands.
	96.2	prototroph	E. Barrio<br>Natural isolate from *Quercus ilex* bark (Castellón, Spain)
K. bulderi	CBS 8638	Prototroph, [rho^{0}] (this study)	**ARS culture Collection (NRRL)<br>Isolated from maize silage
	CBS 8639	Prototroph, [rho^{0}] (this study)	ARS Culture Collection (NRRL)<br>Isolated from maize silage
	NRRL Y-27205	Prototroph, [rho^{-}] (this study)	ARS Culture Collection (NRRL)<br>Isolated from maize silage

*NCYC: National Collection of Yeast Cultures

**Agricultural Research Service culture collection (Northern Regional Research Laboratory)

Supplementary Table 15. Primer sequences for verification of inversion in chromosome VII.

Name	Sequence	Amplicon Size
F1	CCGTTGACCTATGACTGTAC	1787
R1	GATCGCCTGTGGTAAATCTG	
F2	CACCAAGAGCCTGGATTAGA	3041
R2	GCGAATTTAACTTCACCTGG	
F1 + F2		1596
R1 + R2		2363

Supplementary Table 16. Primers for experimental validation of split/incomplete contigs for CBS 8638, CBS8639 and NRRL Y-27205 IPA genome assemblies.

ctg.000016F and ctg000023F of CBS 8639 form Chr VIII		
Chr 8 F	GGAACTTCCCGCATCATTTT	
Chr 8 R	GCTGCTGAGGCTAATGAATT	
Amplicon size	1129	(bp)
ctg.000010F and ctg.000020F in NRRL Y-27205 form Chr III		
Chr 3 F	GTCTAGCACGTAACCTTGGT	
Chr 3 R	CGATAATGATTCGGTTGTTCC	
Amplicon size	1300	(bp)
ctg.00008F and ctg.000024F verification for Chr IV		
Chr 4 F	CTAGGTAATTGAGCAGGTGG	
Chr 4 R	CGGTTACACTACTAACGACC	
Amplicon size	1691	(bp)
Chr VII splits in three contigs		
Starts in ctg. 000027F follow by ctg.000026F finishes in ctg.000006F		
Ch7A F	CCAGCTTCGATATCTTCAGC	
Ch7A R	ACCAACCGTTCGAGTATATC	
Amplicon size	1304	(bp)
Ch7B F	AAGGCTGAACCAACTCCGGA	
Ch7B R	TAGCACGGATTTAGTGTCCCG	
Amplicon size	1107	(bp)
Chr I a + b Start ctg.00001F and ctg.000002F in CBS 8638		
Chr 1A F	GGTACCCTCAGGGTATTCTG	
Chr 1A R	CCGCAGGTTCTAATCAAGCT	
Amplicon	800	(bp)
Chr XII ctg.000019F and ctg.000015F		
Chr 12 F	GGAGCGTAACATCACAGACC	
Chr 12 R	GCCATTAGACCTGGCATTTC	
Amplicon size	834	(bp)
Chr X ctg.000018F and cgt.000017F form chr X		
Chr 10 F	GGTTGCACTGACTTGAGTTC	
Chr 10 R	CGGATGGAATATCCGATGATG	
Amplicon size	789	(bp)

Supplementary Table 17. List of primers employed for PCR validation for chromosome V and I potential translocation.

Name	Sequence
F1	CATCCTTGGAGACGTTAGCTC
R1 A	GACCACACTCCAAACGGTCT
Expected band	8821
F2	GGAGAGAATCTCGCGGTGAG
Expected band	7658
F3	GGAGTGTCGATAGCCATTCTAAC
Expected band	7305
F1'	CGGCTAATGAAATCCTTCCTC
R1B	CTCTACTGCTAAGCCAGGTG
Expected band	5901
R2	GCAGTGATGGCTCAAATGTC
Expected band	4081
VF1	CCAACCACAACGTGTAGTG
VR1	GACCACACTCCAAACGGTCT
Expected band	2556
VF2	GGCACTTGAGACATATGAGC
Expected band	4026

.

Supplementary Figures.

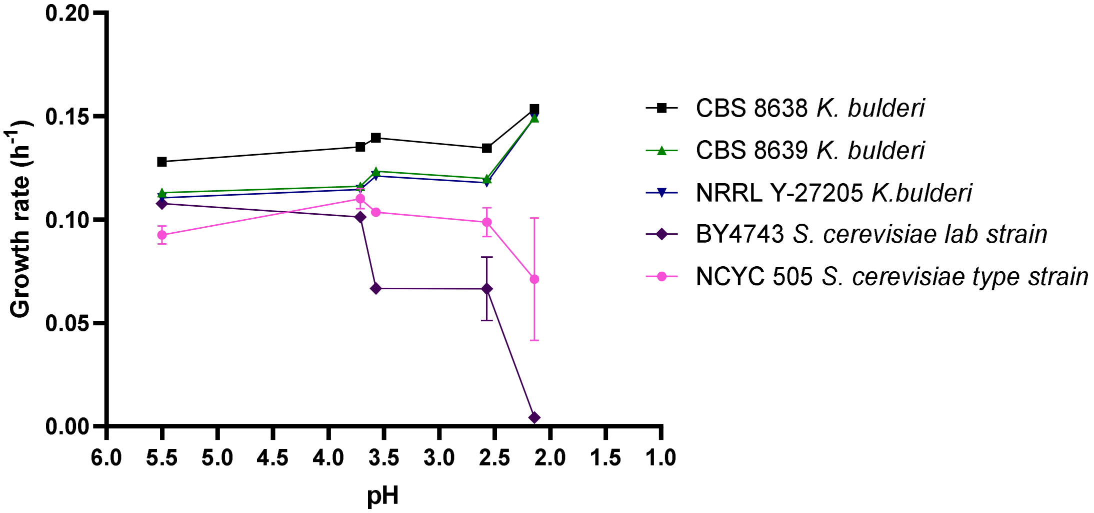

Supplementary Fig. 1: Phenotypic analysis of *K. bulderi* strains CBS 8638, CBS 8639 and NRRL Y-27205 at different pH. Maximum growth rate in minimal SD medium of the three *Kazachstania bulderi* strains compared with *Saccharomyces cerevisiae* lab BY4743 and type strain NCYC 505 from pH 5.5 to 2.1. Error bars represent standard deviation of three biological replicates.

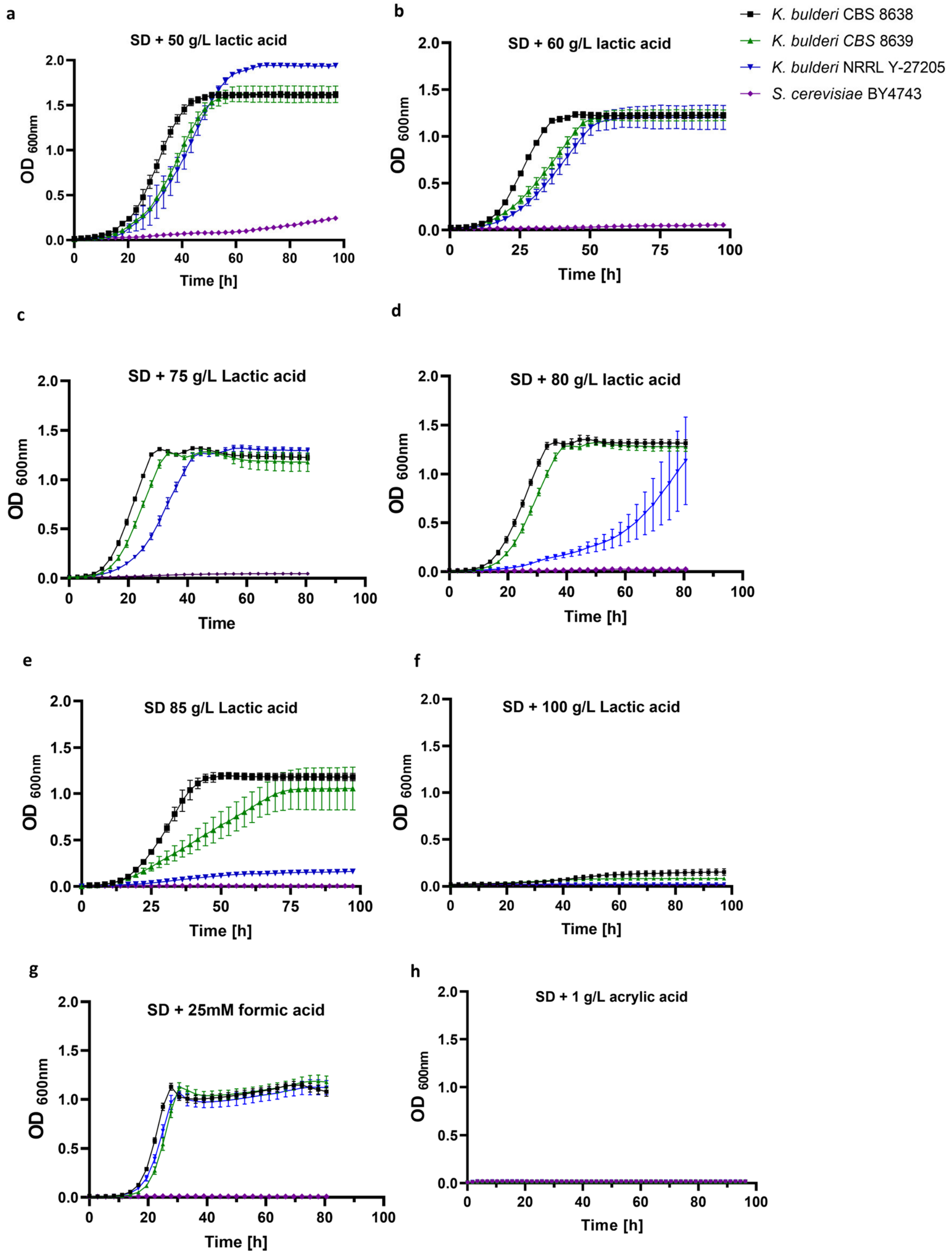

Supplementary Fig. 2: Effect of varied organic acid concentrations on the growth of *K. bulderi* strains. Growth curves of *K. bulderi* strains CBS 8638, CBS 8639 and NRRL Y-27205 and *S. cerevisiae* BY4743 in SD medium containing (a)50g/L, (b) 60g/L, (c) 75g/L, (d) 80g/L, (e) 85g/L and (f) 100g/L of lactic acid at constant pH of 2.5. Growth curves at 25 mM formic acid (g) and 1g/L acrylic acid (h). Error bars represent standard deviation of three biological replicates.

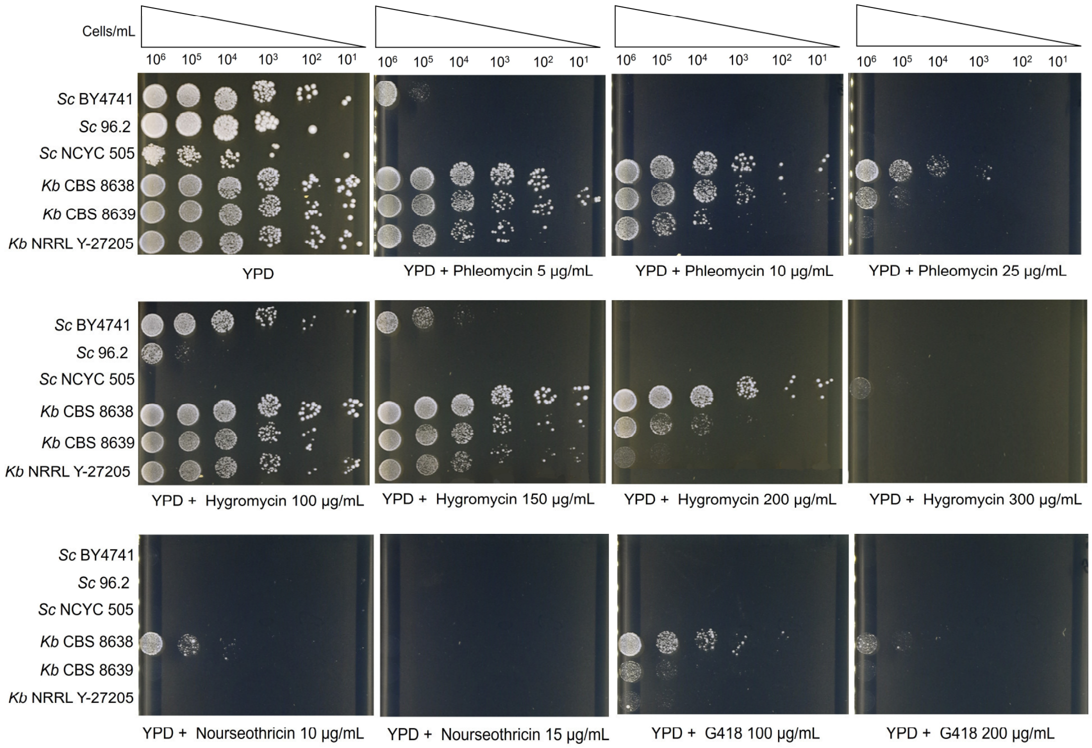

Supplementary Fig. 3: Antimicrobial drug resistance profiling of *K. bulderi* strains. Spot test assay of *K. bulderi* (Kb) strains CBS 8638, CBS 8639 and NRRL Y-27205, and *S. cerevisiae* (Sc) strains BY4741, 96.2 and NCYC 505 in YPD, YPD + phleomycin, YPD + hygromycin B, YPD + nourseothricin and YPD + G418 at different concentrations.

a

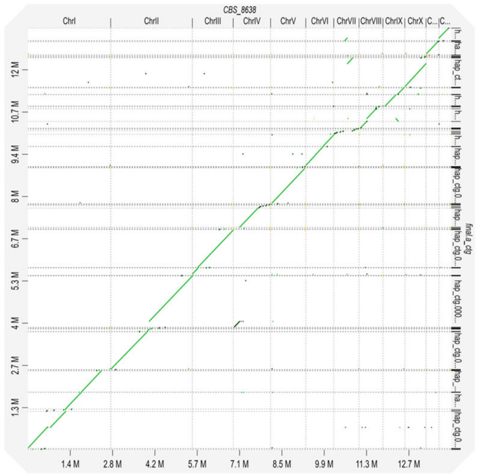

b

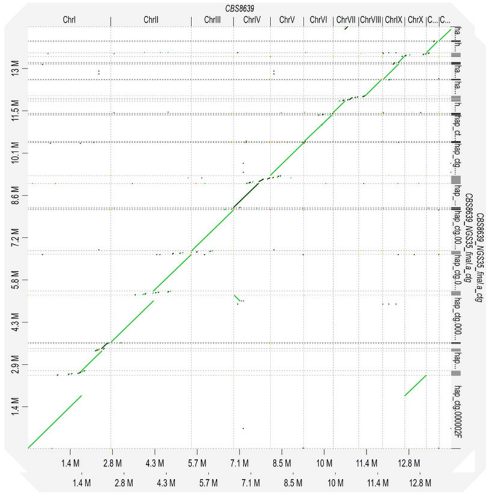

c

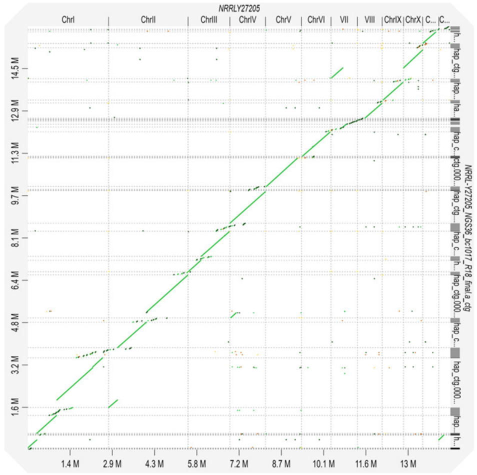

Supplementary Fig. 4: Sequence alignment between principal and alternative assemblies for the three *K. bulderi* strains. Dot plots showing the alignments between principal polished assemblies and their corresponding alternative assemblies in CBS 8638 (a), CBS 8639 (b) and NRRL Y-27205 (c).

a

	CBS 8638		CBS 8639		NRRLY-27205	
	**YGAP**	**Augustus**	**YGAP**	**Augustus**	**YGAP**	**Augustus**
**N° of genes**	5879	6237	5681	6005	5705	6083
**N° of proteins**	5668	6237	5476	6005	5501	6083
**HybridMine**						
**N° of proteins functional annotated**	4563	4524	4507	4468	4496	4462

b

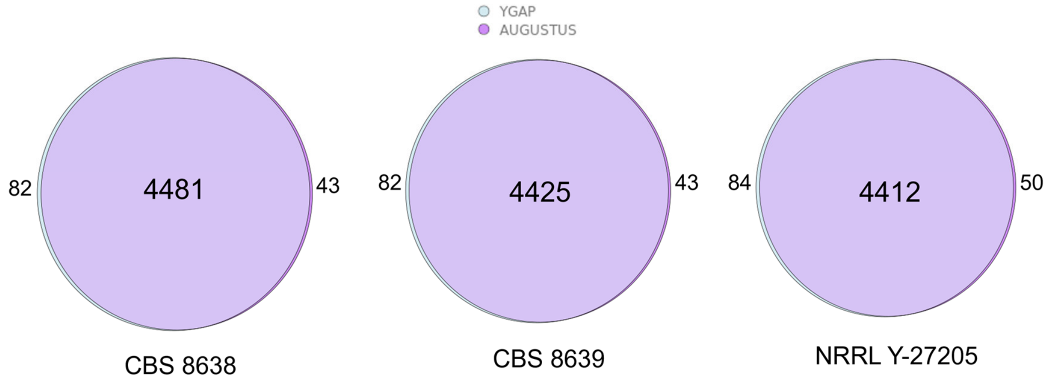

Supplementary Fig. 5: Comparative structural and functional annotation analysis of *K. bulderi* strains. (a) Table containing the number of genes and proteins annotated by YGAP and AUGUSTUS for IPA assemblies of CBS 8638 and CBS 8639, and NRRL Y-27205, and the number of proteins predicted for HybridMine when using *S. cerevisiae* as reference. (b) Proportional Venn diagram with the number of functional annotated proteins predicted for HybridMine in common between the methods for structural annotation, YGAP light blue and AUGUSTUS dark purple, YGAP&AUGUSTUS light purple.

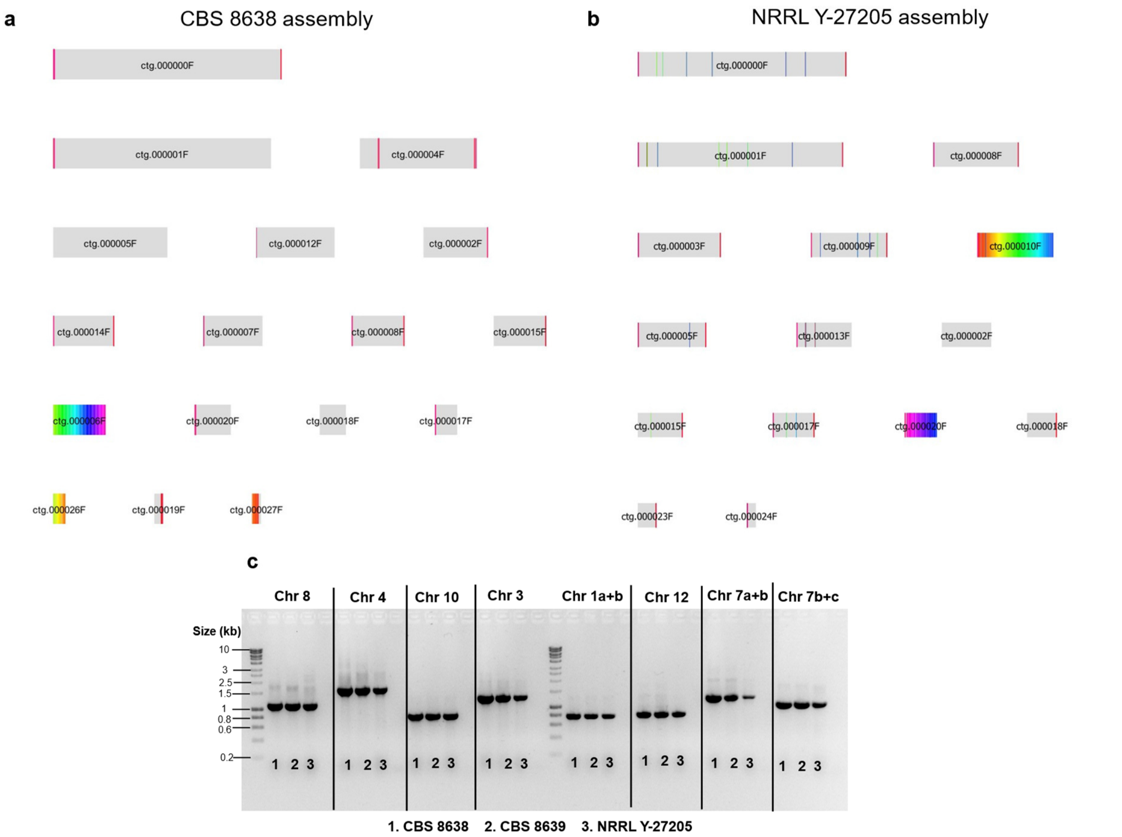

Supplementary Fig. 6: Manual confirmation of the continuity of split contigs by PCR.
(a) Illustration depicting the initial assembly of CBS 8638 (17 contigs) aligned against the initial assembly of CBS 8639 (14 contigs). In CBS 8639, contig ctg.00005F is split into ctg.000027F, followed by ctg.000026F, and concluding with ctg.00006F in CBS 8638. (b) Visual representation of the assembly of NRRL Y-27205 (15 contigs) aligned with CBS 8639. The contig ctg.00006F in CBS 8639 is divided into contig ctg.000010F and ctg.000020F in NRRL Y-27205. Rainbow colours (ranging from red to violet) indicate the continuity of the contigs in CBS 8639 with the best BLAST hits. (c) PCR verification of contig breakpoints for strains CBS 8638, CBS 8639, and NRRL Y-27205 on chromosomes 1, 3, 4, 7, 8, 10, and 12.

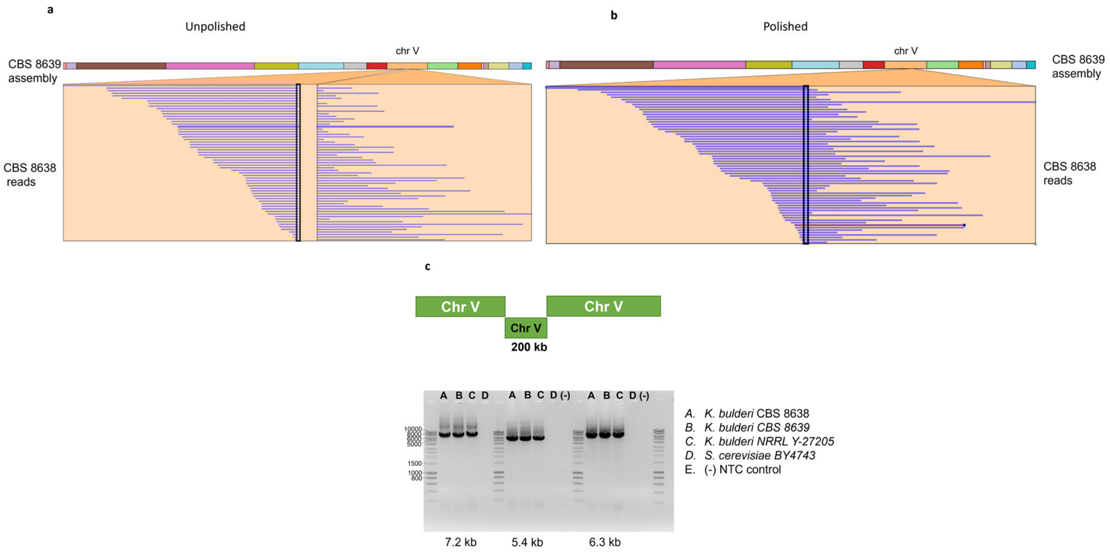

Supplementary Fig. 7: Comparative analysis and PCR confirmation of chromosome V missing region in CBS 8638 strain.

Visual representation of the alignment of CBS 8638 reads against (a) unpolished and (b) polished CBS 8638 genome assembly. (c) PCR-based confirmation of the missing region in chromosome V identified using the alternative assembly approach. Three combinations of primers within the missing region were employed to amplify products in *K. bulderi* CBS 8638, CBS 8639, or NRRL Y-27205 strains. As a specificity control, *S. cerevisiae* BY4743 was used, and a no-template control (NTC) with only water was included.

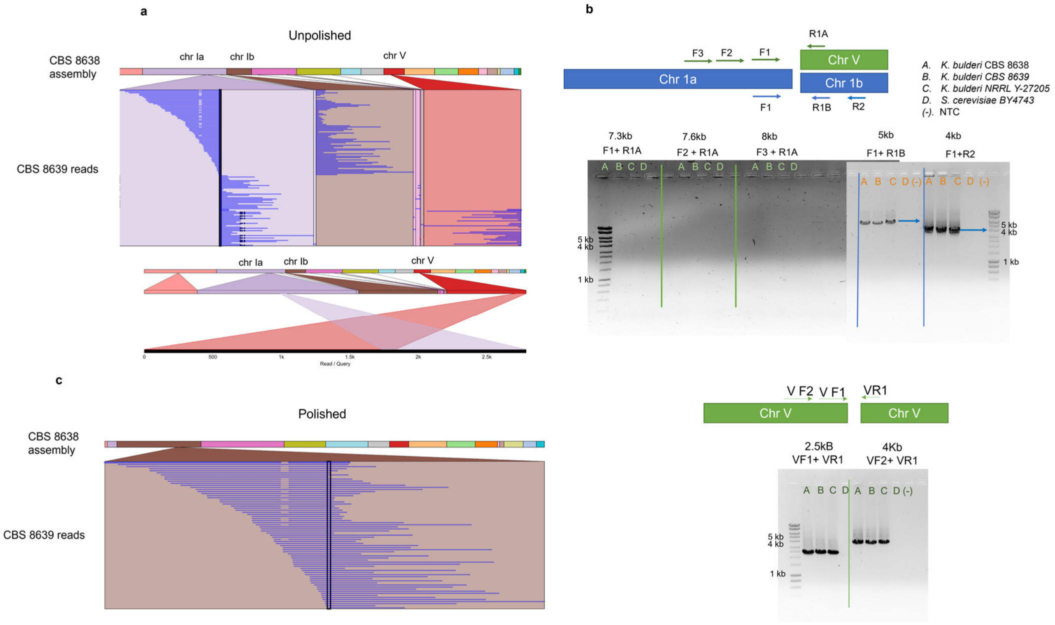

Supplementary Fig. 8: Comparative read coverage analysis and PCR verification of chromosome I and V continuity. (a) Visual representation of the alignment of CBS 8639 reads within the region of chromosome I, which was identified as potentially translocated in chromosome V within the unpolished *K. bulderi* CBS 8638 genome assembly. (b) Illustration depicting the primer locations for PCR verification of the continuity of chromosome I and chromosome V, along with the PCR-based confirmation of their continuity. (c) Alignment of CBS 8639 reads against the CBS 8638 genome assembly post-curation. This step contributed to a refined alignment and improved overall assembly quality.

K. bulderi CBS 8639

Chromosome	Size (Mb)
Chr I	2.79
Chr II	2.72
Chr III	1.42
Chr IV	1.24
Chr V	1.12
Chr VI	0.99
Chr VII	0.86
Chr VIII	0.80
Chr IX	0.75
Chr X	0.72
Chr XI	0.43
Chr XII	0.40

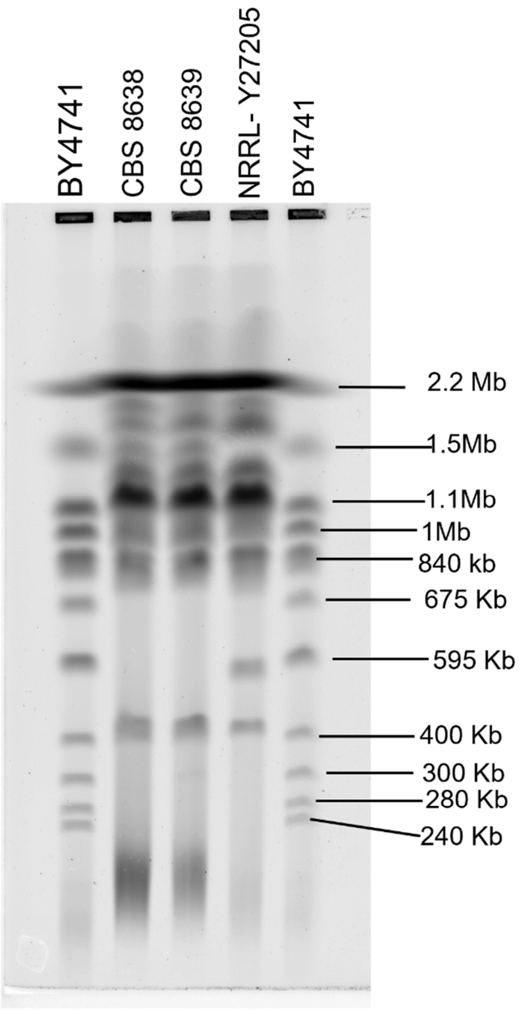

Supplementary Fig. 9: Pulse Field Gel Electrophoresis (PFGE) of chromosomal DNA of all *K. bulderi* strains. Analysis PFGE of chromosomal DNA of *K. bulderi* CBS 8638, CBS 8639 and NRRL Y-27205 strains. The first and the last line contain *S. cerevisiae* BY4741 strain as size marker. The table contains the sizes according to the genome assembly for CBS 8639 strain.

a

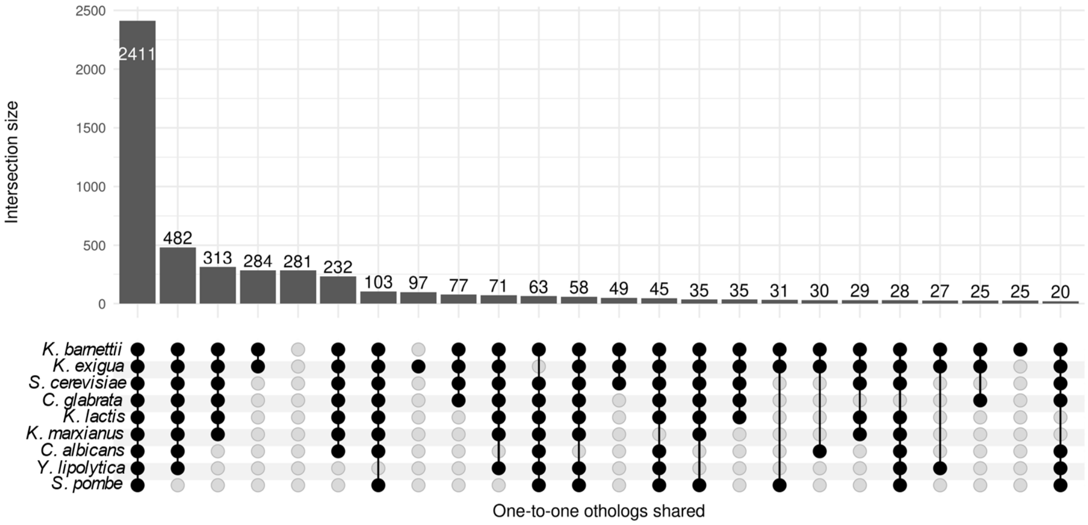

b

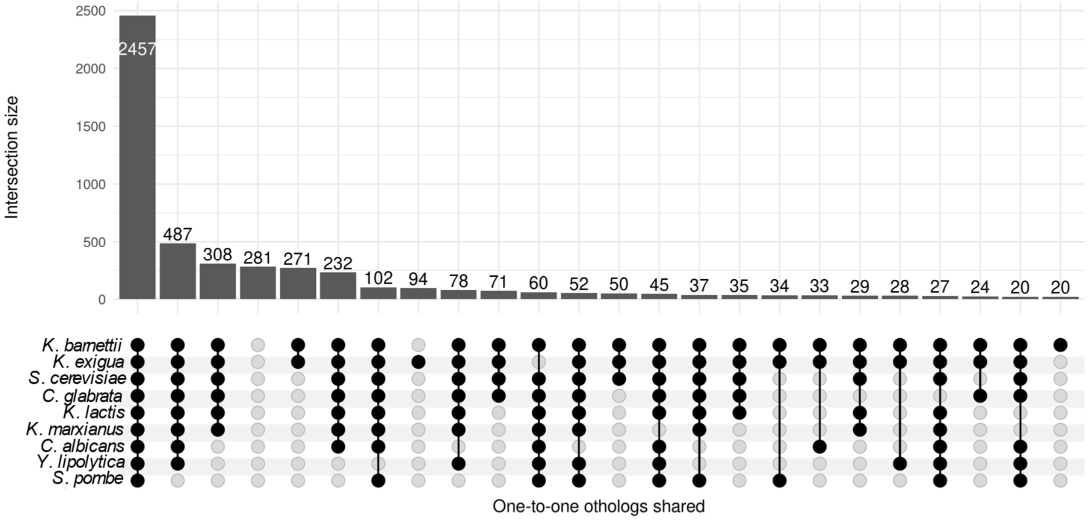

Supplementary Fig. 10: Upset plot showing the number of proteins functionally annotated across *K. bulderi*. Number of proteins annotated in common when using *Saccharomyces cerevisiae*, *Schizosaccharomyces pombe, Candida albicans, Candida glabrata*, *Yarrowia lipolytica*, *Kluyveromyces marxianus, Kluyveromyces lactis, Kazachstania exigua* and *Kazachstania barnettii*, as references genomes in (a) CBS 8638 and (b) NRRL Y-27205 strains in common. Vertical lines across the species represent the proteins annotated in common among them. Individual dots represent the proteins of each species shared only with *K. bulderi*.

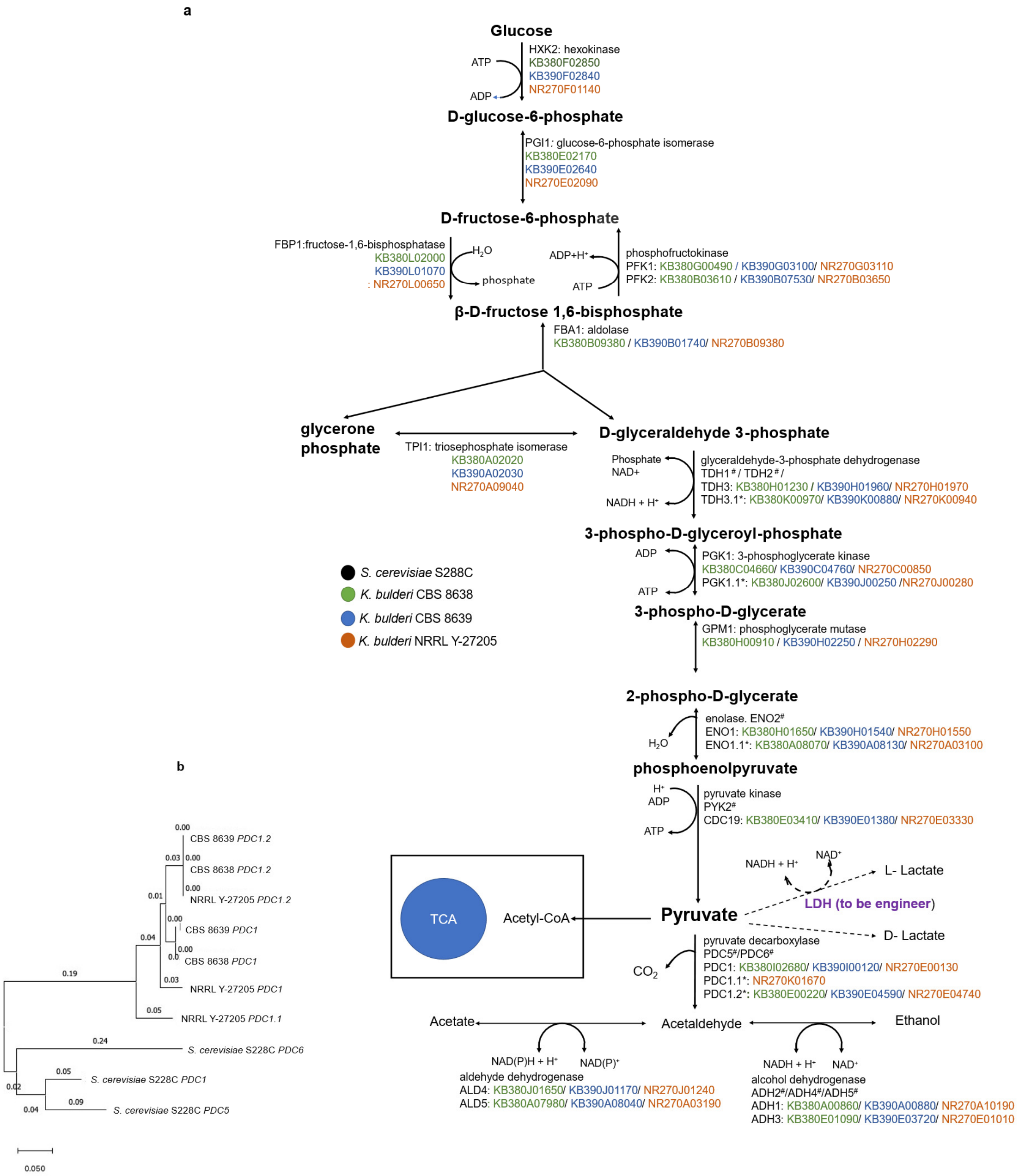

Supplementary Fig. 11: Comparative analysis of key metabolic enzymes in *K. bulderi* and *S. cerevisiae*. (a) Illustration depicting the glycolysis and the pyruvate metabolic pathway from *S. cerevisiae*, *K. bulderi* CBS 8638, CBS 8639, and NRRL Y-27205 highlighted in black, blue, green, and orange, respectively. Paralogs unique to *K. bulderi* strains, distinct from *S. cerevisiae*, are marked with asterisks. Genes exclusive to *S. cerevisiae* and absent in *K. bulderi* strains are indicated by hashtags. Young paralogs are annotated as *PDC1.1* and *PDC1.2*, *ENO1.1, THD3.1* and *PGK1.1*. (b) Phylogenetic tree illustrating the relationship among *PCD* genes and young paralogs *PDC1.1* and *PDC1.2* in *K. bulderi*. The tree is drawn to scale, with branch lengths measured in the number of substitutions per site.

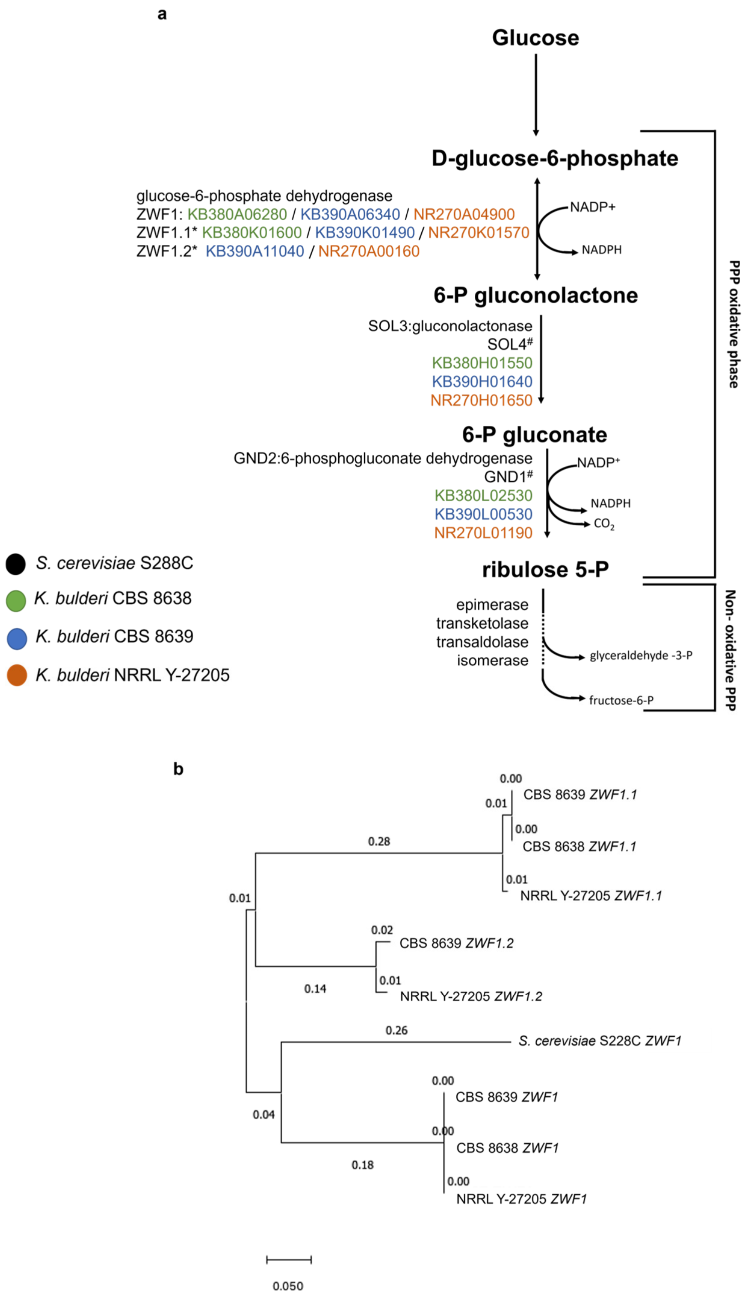

Supplementary Fig. 12: Comparative analysis of pentose phosphate pathway enzymes in *K. bulderi*. (a) Diagram illustrating the enzymes involved in the oxidative phase of the pentose phosphate pathway (PPP) for *S. cerevisiae*, *K. bulderi* CBS 8638, CBS 8639, and NRRL Y-27205. Enzyme names are color-coded as black, blue, green, and orange, respectively. Paralogs unique to *K. bulderi* strains, distinct from *S. cerevisiae*, are marked with asterisks. Genes exclusive to *S. cerevisiae* and absent in *K. bulderi* strains are indicated by hashtags. (b) Phylogenetic tree showcasing the relationship among *ZWF1* genes and their paralogs *ZWF1.1* and *ZWF1.2* within *K. bulderi* strains. The tree is drawn to scale, with branch lengths measured in substitutions per site.

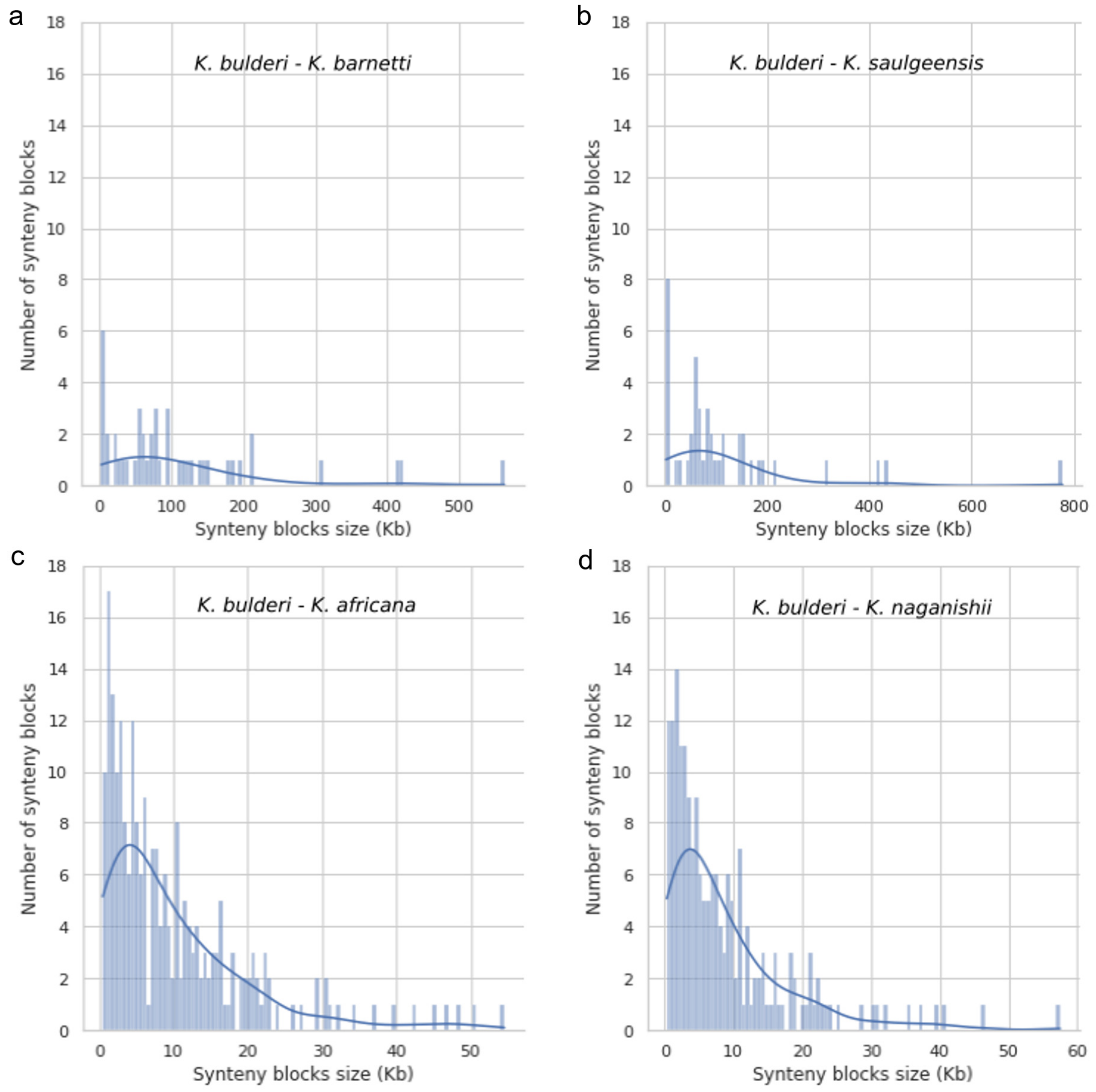

Supplementary Fig. 13: Histogram representing the occurrence of the synteny blocks sizes among *Kazachstania* species. *K. bulderi* CBS 8639 genome is compared to *K. barnettii* (a), *K. saugeensis* (b), *K. africana* (c) and *K. naganishii* (d).

```
>Ya region Ya region undefined product 56227:56339 reverse
ATTGATATGAACATTAAAATATATAGAATATATGTAACAAATATTTCATTTATTGATTAT
TATTATTATTTACTGGTGGTTTAGTACGTGATCGTTTGTTGATAAACTATATA

>Yalpha region Yalpha region undefined product 182200:183687 forward
GTTAATCGGTTCCATAATTTTTGTTCATTTTCATTTGTATGCCAAATCTTGGACAAAATA
TTAGATAATATGTTTTGTTTTAAACCTTTACCATATTGAGAATAATATATTCTAAATGCA
ATAAATCCATTAATTTTCTTATTGGAACATATTTCAAATGGATTCTTCTCGATATTATAA
TTCATAAATGAACCAGATAGATTAACTATCTGATTCATATTTAATGCATCAAAGTTATTT
ACATTGTATATATTATTTATAATATTATATTTTTGATAATATTGTTTATTAATCTCTTGC
TCAATAACTTGGATAATTTTATAATTTGGAATAAATCCATTAAATGGATTAGAACCAACA
ATAAATTCACTAGTAGAATAATTTAATTTATTCTTTTTAATTCGTTTATCATTAATCTTG
ATATTTGAAATTGTCTTGGGTCTCAGAGATTTAGGTTTCTTGTTAGTTAAATTAACTTTA
AATGATGCTTTGGTACTCAACATATTTGGTCTTGTTGTCTTTATTTAGTTAGTTAATATC
ACTTTGTTTGTAAAATAGATGTTTTATATTAGATGCATTTATATACATGGTTTTTTCTAT
AAACCAGTTTCGACCTCAAAATGTTTTCTTTTTTTCCCTCTACTTATCCTTTGCTGCCAA
AAGCAGCAAAGAAAAAAAAAAAATATTAATAGTAGTAGGATAAGAAAATAATTAAAATTTC
TAAGAAAGGGATTATGTTAAATGAATATAATAATAATAAATTATTATATAAAGGAACATA
TATTAAAATTGCATTAACTGAATATTCCTCTTTATTTAGATTCCTAAATAAATATGACAA
CAATGAATAAAATACCAATCCAACATCTACTAAATCCAACATCAAATAAGAGACTTTATA
AATCTGATCTAAAACAGATAAATACAGAATTAATGTATATTTGTTCTTCATTACCTGAAG
GAATATTTCAAAATATAAAAGATGTTAAAATACATCTTCATGATATTGTAACAAAACTAG
GAATAATAAAAGGTCAAGGTAAATTAGATAATGAGGAAAGATATTTAATTAAGATATCAT
TCCAATTGGCAACAATAGTTGCCAACTTCCTGAAAAGTGTTGAGGAAGAAAAGAAACCAA
GCAAAGATTCAAAAAAGAAAAAGAAACAAGGAAAATCTTCAAAAGAAGAAGAAGTAGTAT
TTAATGTTGTAACACAAAATATGATGAATATGGATAATGTAAAACAAAATTCATTTAGAG
GTCACAGATTCTCAAAACAAAATGTGGATGTATTAGAAGAATGGTATAGTATTCATAAAC
ATAAACCATATTTAGATAAGAAAAGTATAGAAAGGTTACAAAGTCAAACACAATTATCGA
GAACACAAATTAAAAATTGGGTATCAAACAAGAGAAGGAAAGAGAAAACTGTACAAGTGT
CATCAGAGATTCTACAACTAATAAACGGTAAATAAAGAAAAGCGCAAG
```

Supplementary Fig. 14: DNA sequences of the Y regions of *MAT* locus. Sequences of Ya or Yalpha were used in BLASTN searches to annotate *MATalpha* and *MAT*a genes in the three *K. bulderi* strains.

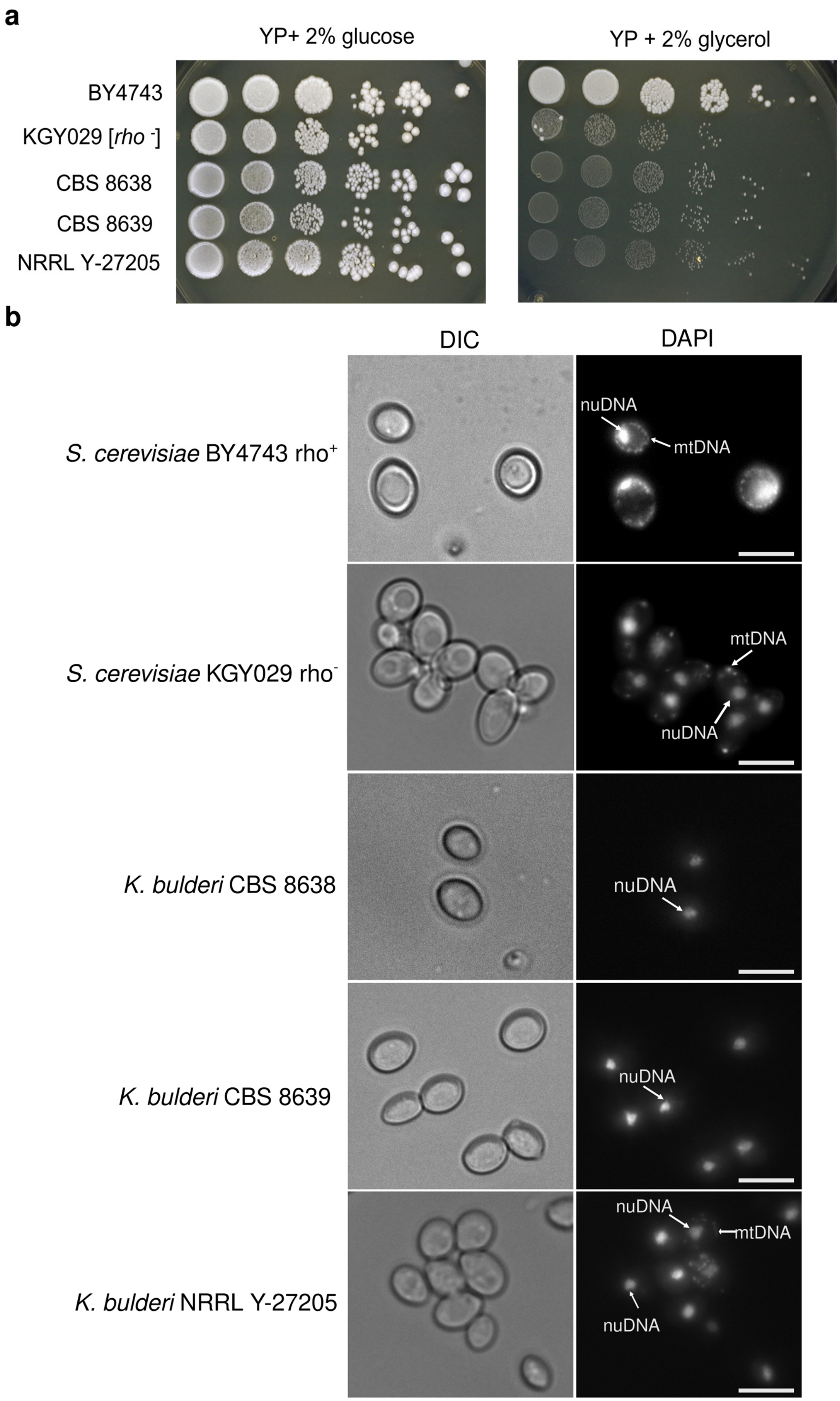

a
YP+ 2% glucose
YP + 2% glycerol
BY4743
KGY029 [rho -]
CBS 8638
CBS 8639
NRRL Y-27205
b
DIC
DAPI
S. cerevisiae BY4743 rho+
nuDNA
mtDNA
S. cerevisiae KGY029 rho-
mtDNA
nuDNA
K. bulderi CBS 8638
nuDNA
K. bulderi CBS 8639
nuDNA
K. bulderi NRRL Y-27205
nuDNA
mtDNA
nuDNA

Supplementary Fig. 15: Respiration capability and mitochondrial DNA staining of *K. bulderi.* (a) Spot test assay for CBS 868, CBS 8639 and NRRL Y-27205 *K. bulderi* strains on YP+ 2% glucose and YP+ 2% glycerol. Comparison is made against *Saccharomyces cerevisiae* BY47413 (WT) and *COX2*-mutant rho $^{-}$ strain (KGY029) that contains a mutation on *COX2* gene affecting mtDNA and avoid its grow in glycerol. (b) DAPI staining of *K. bulderi* CBS 8638, CBS 8639 and NRRL Y-27205 strains along with *S. cerevisiae* BY4743 and KGY029. Cells were observed under differential interphase contrast (DIC) and after DAPI staining. White arrows point to mitochondrial DNA (mtDNA) and nuclear DNA (nuDNA). The mtDNA in KGY029 shows a network of punctuate dots, a phenotype common in rho^{-} strains. Scale bars: 5µm

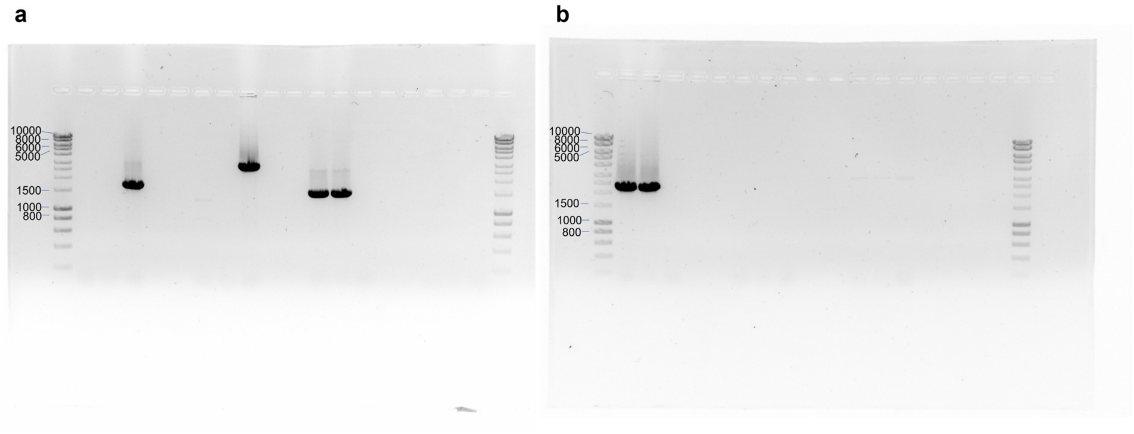

Supplementary Fig. 16: Uncropped gel images corresponding to Fig. 4 in the main manuscript. (a) Visualization of PCR bands using the primers couples F1+R1, F1+ F2, and F2+R2 or (b) R1+R2.

a

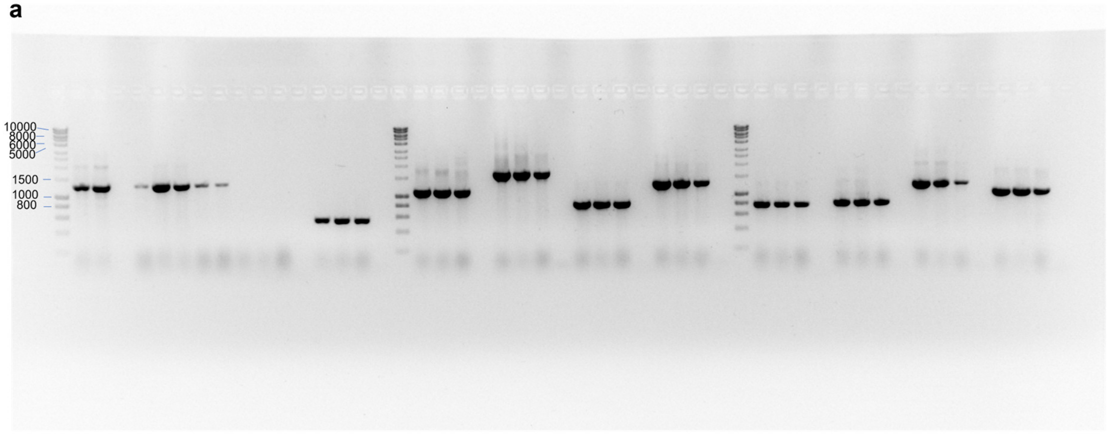

b

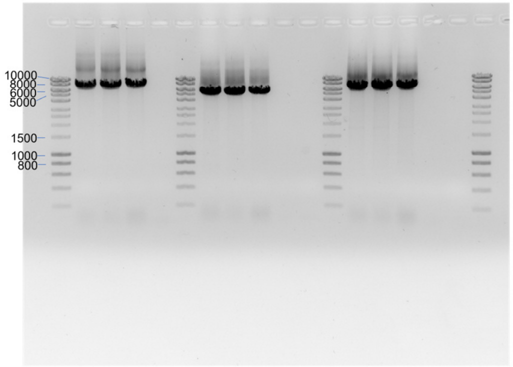

Supplementary Fig. 17. Uncropped version of gel images presented in the Supplementary Figures 6 and 7. (a) Gel corresponding to Fig.6c and (b) Supplementary Fig. 7c.

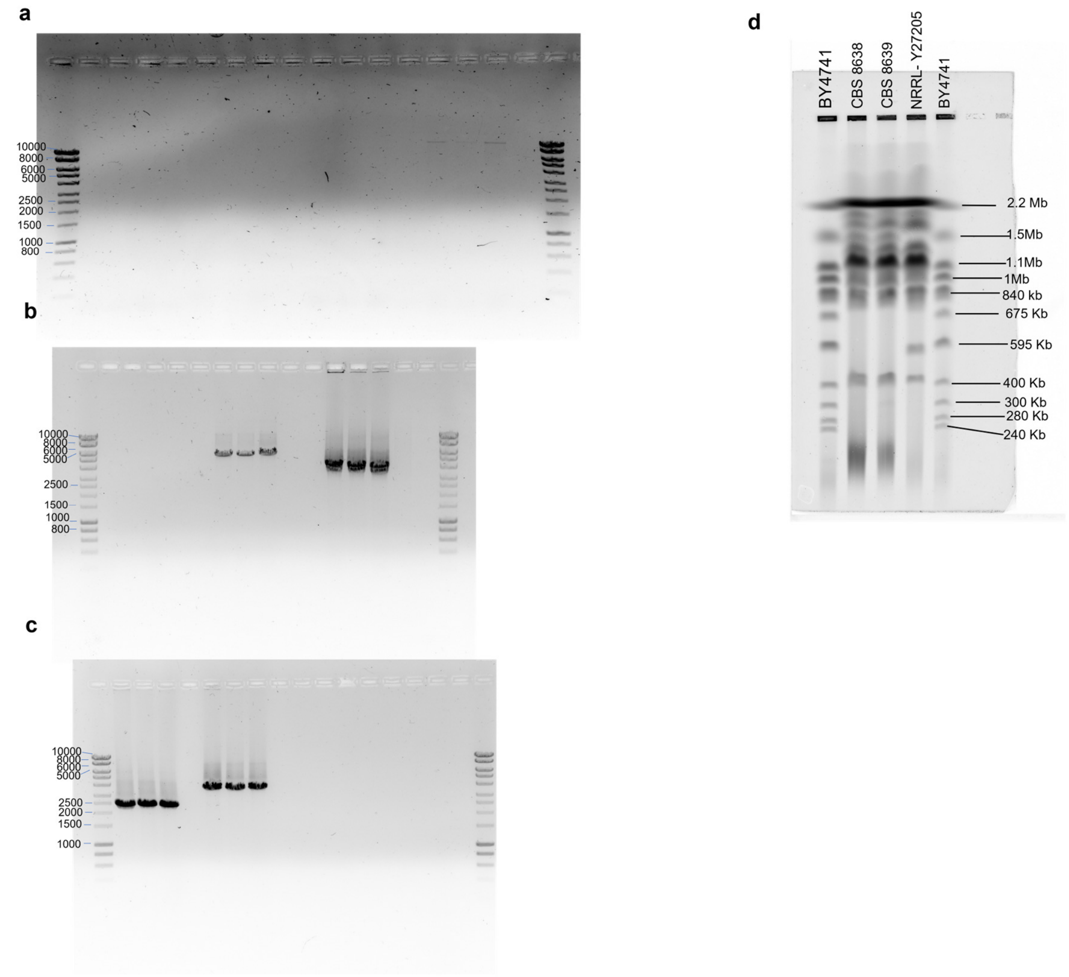

Supplementary Fig. 18: Uncropped version of gel images presented in the Supplementary Figures 8 and 9. Visualization of PCR bands presented in Supplementary Fig. 8b corresponding to chromosome I using the primer couples (a) R1A+ F1, R1A+ F2 and R1A+ F3 or (b) F1+ R1B and F1+R2; and to chromosome V using the primer couples (c) VR1+ VF1 and VR1+ VF2. (d) PFGE gel image shown in Supplementary Fig. 9.

Supplementary Notes

Supplementary Note 1: Manual curation: confirmation of contigs continuity

To resolve incomplete/split contigs in the IPA assemblies deep comparison between the initial assemblies of the three *K. bulderi* were carried out by aligning CBS 8639 genome assembly against CBS 8638 and NRRL Y-27205. All *de novo* assembly graphs were visualized using Bandage v0.8.1 [1] and the alignment showed single contigs that have been assembled separated on two or even three different contigs (Supplementary Table 6). For instance, contig ctg.00005F of CBS8639 was split in contig ctg.00006F, ctg.00026F, ctg.00027F in CBS 8638 initial assembly (Supplementary Figure 5).

Candidate sequences for long PCR validation were extracted by selecting the regions at the edge of each the split/incomplete contig. We validated the continuity of chromosome 1, 3, 4, 8, 10 and 12 that were broken in two different contigs and chromosome 7 that was split in three different contigs in CBS 8638 assembly (Supplementary Figure 5c). A list of primers with a detailed explanation of potential split contigs and amplicon sizes are listed in Supplementary Table 16.

The continuity of the contigs were further verified by mapping the CCS reads of each strain against each *de novo* assembly by Minimap2 v2.24 [2,3]. We identified lineal read coverage and resolve potential contigs translocated or misplaced by the IPA assembler by visualizing the alignment using Ribbon [4].

Supplementary Note 2: Manual curation: identification of missing coverage around genome assemblies.

Approximately 200,000 base pairs (bp) of coverage were absent from chromosome V (Supplementary Fig. 6). Although this region was present in CBS 8638, it was notably absent in both CBS 8639 and NRRL Y-27205. In order to ascertain the presence of this DNA segment across all strains, we employed alternative haplotypes to locate the missing sequence. To validate the absence of the region, we conducted PCR and re-mapped the reads to ensure continuity (Supplementary Fig.6). Our investigation resulted in the recovery of approximately 56 genes situated within the missing segment.

The chromosome-like polished assembly led to a reduction in the number of mismatches per 100 kb, dropping from 706.3 to 695.96 in CBS 8638 and from 842.98 to 824.63 in NRRL Y-27205

Supplementary Note 3: Manual curation: validation of chromosome I and V in CBS 8638 genome assembly.

Through the mapping of CBS 8638 and CBS 8639 reads against the CBS 8638 assembly, we identified non-continuous coverage in chromosomes I and V. Specifically, we observed a disruption in the region at the end of chromosome I, where reads originating from chromosome V interrupted the continuity (Supplementary Fig. 7a). This pattern resembled translocation events.

To investigate whether this interruption indicated a translocation or a misassembly, Various primer combinations were employed to amplify products specific to CBS 8638, CBS 8639, or NRRL Y-27205 strains. S. cerevisiae BY4743 was utilized as a negative control. Primers were located upstream and downstream of the coverage breakpoint (Supplementary Fig. 7b). The resulting PCR products consistently supported the identified misassembly and excluded the possibility of translocation (Supplementary Fig. 7b). Primers are listed in Supplementary Table 17.

This outcome effectively eliminates the possibility of a translocation and underscores the susceptibility of assembly algorithms to misassemblies. It emphasizes the vital role of manual curation in achieving a high-quality *de novo* genome assembly. Upon correcting the assembly and aligning the reads to the refined CBS 8638 assembly, we successfully achieved continuous coverage for chromosome V (Supplementary Fig. 7c).

Supplementary references

1. Wick, R. R., Schultz, M. B., Zobel, J. & Holt, K. E. Bandage: interactive visualization of de novo genome assemblies. *Bioinformatics* **31**, 3350–3352 (2015).

2. Li, H. Minimap2: pairwise alignment for nucleotide sequences. *Bioinformatics* **34**, 3094–3100 (2018).

3. Li, H. New strategies to improve minimap2 alignment accuracy. *Bioinformatics* **37**, 4572–4574 (2021).

4. Nattestad, M., Aboukhalil, R., Chin, C. S. & Schatz, M. C. Ribbon: intuitive visualization for complex genomic variation. *Bioinformatics* **37**, 413–415 (2021).
