# Supplementary material for: High quality de novo genome assembly of the non-conventional yeast Kazachstania bulderi describes a potential low pH production host for biorefineries
Source: Commun Biol. 2023 Sep 7;6:918. doi: 10.1038/s42003-023-05285-0 (PMC10484914; doi:10.1038/s42003-023-05285-0)
Supplement: Supplementary file 2 — Description of Additional Supplementary Files [file 42003_2023_5285_MOESM2_ESM.pdf]

Description of Additional Supplementary Files

File name: Supplementary Data 1
Description: Group of homologs for CBS 8638, CBS 8639 and NRRL Y-27205 K. bulderi strains predicted by HibridMine.

File name: Supplementary Data 2
Description: Number of proteins functionally annotated by HybridMine in K. bulderi using a range of reference organisms.

File name: Supplementary Data 3
Description: Predicted GO terms by DeepFRI for Molecular Function (MF), Biological Process (BP) and Cellular Components (CC) GO terms and EC numbers for the potential specific K. bulderi proteins.

File name: Supplementary Data 4
Description: Number of genes within homozygous regions in CBS 8638, CBS 8639 and NRRL Y-27205 K. bulderi strains.

File name: Supplementary Data 5
Description: Gene list and sizes of the synteny blocks between K. bulderi CBS 8639 other Kazachstania assembled in chromosomes.
