# Supplementary material for: High quality de novo genome assembly of the non-conventional yeast Kazachstania bulderi describes a potential low pH production host for biorefineries
Source: Commun Biol. 2023 Sep 7;6:918. doi: 10.1038/s42003-023-05285-0 (PMC10484914; doi:10.1038/s42003-023-05285-0)
Supplement: Supplementary file 8 — Reporting Summary [file 42003_2023_5285_MOESM8_ESM.pdf]

nature portfolio

Corresponding author(s): Daniela Delneri

Last updated by author(s): Aug 15, 2023

Reporting Summary

Nature Portfolio wishes to improve the reproducibility of the work that we publish. This form provides structure for consistency and transparency in reporting. For further information on Nature Portfolio policies, see our Editorial Policies and the Editorial Policy Checklist.

Statistics

For all statistical analyses, confirm that the following items are present in the figure legend, table legend, main text, or Methods section.

n/a	Confirmed	
☐	☒	The exact sample size (*n*) for each experimental group/condition, given as a discrete number and unit of measurement
☐	☒	A statement on whether measurements were taken from distinct samples or whether the same sample was measured repeatedly
☒	☐	The statistical test(s) used AND whether they are one- or two-sided<br>*Only common tests should be described solely by name; describe more complex techniques in the Methods section.*
☒	☐	A description of all covariates tested
☒	☐	A description of any assumptions or corrections, such as tests of normality and adjustment for multiple comparisons
☐	☒	A full description of the statistical parameters including central tendency (e.g. means) or other basic estimates (e.g. regression coefficient) AND variation (e.g. standard deviation) or associated estimates of uncertainty (e.g. confidence intervals)
☒	☐	For null hypothesis testing, the test statistic (e.g. *F*, *t*, *r*) with confidence intervals, effect sizes, degrees of freedom and *P* value noted<br>*Give P values as exact values whenever suitable.*
☒	☐	For Bayesian analysis, information on the choice of priors and Markov chain Monte Carlo settings
☒	☐	For hierarchical and complex designs, identification of the appropriate level for tests and full reporting of outcomes
☒	☐	Estimates of effect sizes (e.g. Cohen's *d*, Pearson's *r*), indicating how they were calculated

Our web collection on statistics for biologists contains articles on many of the points above.

Software and code

Policy information about availability of computer code

Data collection	PacBio: Sequel User Interface Software UI v8.0.0.84541, Instrument Control Software ICS v8.0.0.84541 and Primary Analysis Software PA v8.0.0.84541.
Data analysis	PacBio's open-source SMRT Link 8.0 software, Improved Phased Assembly (IPA v1.8.0) available at https://github.com/PacificBiosciences/pbipa.git, HybridMine v4.0 Available at https://github.com/Sookie-S/HybridMine, HiFiasm assembly tool, Bandage v0.8.1, minimap2 v2.24, Nanopore analysis tools https://nanoporetech.com/resource-centre/analysis-tools-nanopore-data, Ribbon interactive online visualization tool available at http://genomeribbon.com, BLAST v2.12.0, QUAST v5.0.114, BUSCO v5.3.2, MEGA11 v11.0.11, Circa available at http://omgenomics.com/circa, Yeast Genome Annotation Pipeline (YGAP) online version available at http://wolfe.ucd.ie/annotation/, AUGUSTUS v3.4.0, AlphaFold v2.1.1, DeepFRI v1.0.0, Burrows–Wheeler Aligner (BWA) v0.7.17-r1188, pbmm2 v1.10.0 available at https://github.com/PacificBiosciences/pbmm2, SAMtools v1.10, BCFtools v1.10.2, DeepVariant v1.5.0 ,D-GENIES online tool available at http://dgenies.toulouse.inra.fr/, MUMmer v3.0, ShinySyn available at https://github.com/obenno/ShinySyn, Ocular Image Acquisition Software v2.0, ImageJ available at imagej.nih.gov/ij/download/.

For manuscripts utilizing custom algorithms or software that are central to the research but not yet described in published literature, software must be made available to editors and reviewers. We strongly encourage code deposition in a community repository (e.g. GitHub). See the Nature Portfolio guidelines for submitting code & software for further information.

Data

Policy information about availability of data

All manuscripts must include a data availability statement. This statement should provide the following information, where applicable:

- Accession codes, unique identifiers, or web links for publicly available datasets
- A description of any restrictions on data availability
- For clinical datasets or third party data, please ensure that the statement adheres to our policy

The genome assemblies of CBS 8638, CBS 8639 and NRRL Y-27205 strains generated in this study have been deposited in the National Library of Medicine (https://www.ncbi.nlm.nih.gov/) database under the accession numbers SAMN32971551, SAMN32971552, SAMN32971553, respectively. The Bioproject number is PRJNA929900. Data are also available from the corresponding authors upon request.

Human research participants

Policy information about studies involving human research participants and Sex and Gender in Research.

Reporting on sex and gender	n/a
Population characteristics	n/a
Recruitment	n/a
Ethics oversight	n/a

Note that full information on the approval of the study protocol must also be provided in the manuscript.

Field-specific reporting

Please select the one below that is the best fit for your research. If you are not sure, read the appropriate sections before making your selection.

☒ Life sciences ☐ Behavioural & social sciences ☐ Ecological, evolutionary & environmental sciences

For a reference copy of the document with all sections, see nature.com/documents/nr-reporting-summary-flat.pdf

Life sciences study design

All studies must disclose on these points even when the disclosure is negative.

Sample size	K. bulderi CBS 8638, CBS8639 and NRRL Y-27205 strains. Whole genome sequencing and assembly of these two strains were performed.
Data exclusions	No data was excluded
Replication	Genome sequence data of three strains of K. bulderi strains
Randomization	No samples were randomized, as whole genome sequencing was performed here.
Blinding	No samples were randomized, as whole genome sequencing was performed here

Reporting for specific materials, systems and methods

We require information from authors about some types of materials, experimental systems and methods used in many studies. Here, indicate whether each material, system or method listed is relevant to your study. If you are not sure if a list item applies to your research, read the appropriate section before selecting a response.

Materials & experimental systems

n/a	Involved in the study
☒	☐ Antibodies
☒	☐ Eukaryotic cell lines
☒	☐ Palaeontology and archaeology
☒	☐ Animals and other organisms
☒	☐ Clinical data
☒	☐ Dual use research of concern

Methods

n/a	Involved in the study
☒	☐ ChIP-seq
☒	☐ Flow cytometry
☒	☐ MRI-based neuroimaging
